# Non-Volatile Metabolic Profiling and Regulatory Network Analysis in Fresh Shoots of Tea Plant and Its Wild Relatives

**DOI:** 10.3389/fpls.2021.746972

**Published:** 2021-10-01

**Authors:** Chen-Kai Jiang, Zhi-Long Liu, Xuan-Ye Li, Sezai Ercisli, Jian-Qiang Ma, Liang Chen

**Affiliations:** ^1^Key Laboratory of Tea Biology and Resources Utilization, Ministry of Agriculture and Rural Affairs, Tea Research Institute of the Chinese Academy of Agricultural Sciences, Hangzhou, China; ^2^State Key Laboratory for Quality and Safety of Agro-Products, Zhejiang Academy of Agricultural Sciences, Hangzhou, China; ^3^Lishui Academy of Agricultural and Forestry Sciences, Lishui, China; ^4^Department of Horticulture, Faculty of Agriculture, Ataturk University, Erzurum, Turkey

**Keywords:** correlation, fresh shoots, network, non-volatile metabolites, tea plant

## Abstract

There are numerous non-volatile metabolites in the fresh shoots of tea plants. However, we know little about the complex relationship between the content of these metabolites and their gene expression levels. In investigating this, this study involved non-volatile metabolites from 68 accessions of tea plants that were detected and identified using untargeted metabolomics. The tea accessions were divided into three groups from the results of a principal component analysis based on the relative content of the metabolites. There were differences in variability between the primary and secondary metabolites. Furthermore, correlations among genes, gene metabolites, and metabolites were conducted based on Pearson's correlation coefficient (PCC) values. This study offered several significant insights into the co-current network of genes and metabolites in the global genetic background. Thus, the study is useful for providing insights into the regulatory relationship of the genetic basis for predominant metabolites in fresh tea shoots.

## Introduction

Tea plants have a long history of cultivation, processing, and consumption worldwide because they contain special metabolites, specifically secondary metabolites. The metabolites in tea plants play a role in abiotic and biotic stresses during their growth and development. Furthermore, these metabolites are retention substances or precursors that transform other quality compounds in tea. Therefore, metabolites in the fresh shoots of tea plants, as substance bases, contribute to the flavor and health-enhancing functions of drinkable tea. Many studies have been conducted to explore the phytochemical compounds in the leaves of tea plants. These metabolites can be divided into volatile and non-volatile compounds based on their boiling points. Non-volatile compounds account for 99.97–99.99% of the total dry tea weight (Kim et al., 2016), among which flavonoids, theanine, and caffeine have attracted extensive attention during the last few decades. It is important to improve our understanding of the regulatory network of metabolites. Firstly, it is helpful in altering the expression of key genes and the content of metabolites through cultivation measures. Secondly, it forecasts the quality and yield of plants based on metabolite traits that are easy to test. Thirdly, it allows the control of metabolic flow by improving or inhibiting the activity of major enzymes in metabolic engineering. However, most previous studies have focused on the relationships between genes and metabolites. Therefore, the relationship between metabolites remains unclear and requires further probing to understand the regulatory mechanism underlying their relationship.

Previous studies have investigated the effects of an ambient environment, including light, temperature, elevation, and cultivation measures, on the metabolites of tea (Kfoury et al., 2018; Liu et al., 2018). For example, high elevation teas contain statistically sweeter, floral, honey-like compounds as opposed to low elevation tea, which contains statistically greener, herbal, hay-like, bitter compounds (Kfoury et al., 2018). The reduction in flavonols and catechins in shading tea plants was mainly modulated through the downregulation of biosynthetic genes and transcription factors associated with flavonoid biosynthesis caused by reduced UV-B radiation (Liu et al., 2018). Other studies have focused on the regulation of compound biosynthesis by certain genes or metabolic differences in certain varieties (Cao et al., 2020; Li et al., 2021; Zheng et al., 2021). For instance, 13 metabolites were associated with the zigzag-shaped morphology of tea plants (Cao et al., 2020). Five miRNAs may play important roles in regulating the biosynthesis of flavor compounds, including linalool, geraniol, and 2-phenylethanol, in the different tissues of tea plants (Li et al., 2021). A large number of metabolites related to light protection were found to significantly accumulate in the albino tea cultivar, including flavones, anthocyanins, flavonols, flavanones, vitamins and their derivatives, and polyphenols and phenolamides (Zheng et al., 2021). Furthermore, untargeted metabolomic analyses detected 129 and 199 annotated metabolites that were differentially accumulated in different tea groups, and signature metabolites were identified (Yu et al., 2020). However, few studies have investigated the metabolic variations of tea plants in terms of their global genetic background and elucidated the relationship among metabolites. The compound biosynthesis in tea plants is tightly regulated by internal regulatory factors and environmental cues. Genes and metabolites are two internal factors that regulate the expression levels of downstream genes and the content of downstream metabolites. Thus, it is necessary to investigate the regulatory networks among gene–gene, gene–metabolite, and metabolite–metabolite.

Over the past decades, metabolomics has been widely applied in the identification and quantification of metabolites in plants. Ultra-performance liquid chromatography (UPLC) coupled with mass spectrometry (MS) is a powerful tool for simultaneously detecting 100s of non-volatile compounds in tea. In this study, one bud and two leaves (two and a bud) were harvested from tea plants in the first flush of spring in Hangzhou, China. By using untargeted metabolomics integrated with transcriptomics, we performed comprehensive metabolic profiling and verified the co-current network of genes and metabolites associated with the predominant components in fresh tea shoots. Our results provided insights into the genetic basis of important secondary metabolites, such as catechins, caffeine, and theanine, and will be helpful in accelerating genetic improvement and tea breeding in the future.

## Materials and Methods

### Plant Materials

The samples were collected from 68 accessions of tea plants in the section *Thea* (L.) Dyer, genus *Camellia* L., namely, 42 accessions of *C. sinensis* (L.) O. Kuntze var. *sinensis*; 17 accessions of *C. sinensis* var. *assamica* (Masters) Kitamura; 6 accessions of *C. sinensis* var. *pubilimba* Chang; 1 accession each of *C. tachangensis* F. C. Zhang, *C. taliensis* (W. W. Smith) Melchior, and *Camellia* sp. (Supplementary Table 1). Standard two and a bud in the first round were harvested from at least 10 individual tea plants from March 16 to April 30, 2019. The growth vigor of all the tea plants was similar under the same cultivation conditions and agricultural practices planted in the China National Germplasm Hangzhou Tea Repository at the Tea Research Institute, Chinese Academy of Agricultural Sciences in Hangzhou, China. Samples were treated with liquid nitrogen (LN) immediately after collection from the tea plants and were stored at −80°C until they were freeze-dried.

### Sample Extraction

We added 10 ml of 70% methanol with an internal standard (0.025 mg/ml of sulfacetamide and 0.075 mg/ml of tolbutamide) to 200 mg (±0.1 mg) of tea powder. The mixture was extracted in an ultrasonic unit (Branson 5510, Branson Ultrasonics Co., Ltd., USA) at 40 for 30 min at 80 W. The supernatants were filtered through a 0.22-μm filter membrane after stewing at 4°C in the dark for 2 h. The extracts were stored at −80°C until injection. Quality control (QC) was prepared by pooling 100 μl of the extract from all samples.

### Liquid Chromatography–Mass Spectrometry Conditions

Standards and metabolites were detected using a UPLC (Thermo Scientific Dionex Ultimate 3000, Thermo Fisher Scientific, Waltham, MA, USA)-Q-Orbitrap (Thermo Scientific Q Exactive, Thermo Fisher Scientific, Waltham, MA, USA) with an Agilent SB-AQ C18 column (1.8 μm, 2.1 mm × 100 mm, Agilent Technologies, Santa Clara, CA, USA). Solvents A and B were water containing 0.1% formic acid and acetonitrile. The injection was 2 μl. The flow rate was 0.3 ml/min. The column temperature was set to 40°C. The gradient evolution program was as follows: 0–6 min, 5–20% B; 6–10 min, 2,095% B; 10–11.5 min, 95% B; and 11.5–15 min, 95–5% B. The MS operation parameters were as follows: an ion source, electrospray ionization; source temperature, 550°C; normalized collision energy, 15, 30, and 60; isolation window, 4 m/z (mass-to-charge ratio); loop count, 10; dynamic exclusion, 10.0 s; positive and negative modes with electron spray ionization at capillary voltages of 3.5 and 3.2 kV, respectively; and the temperatures of drying gas and aux gas were 320 and 350°C, respectively. The mass range was set from 70 to 1,000 at a resolution of 70,000, and the top 10 peak areas were selected. Samples in the same year were run in the same experiment, and several technical replicates of a QC were distributed across every 10 samples to reduce the influence of intensity drifts.

### Metabolic Data Processing

The m/z, retention time (RT), characterized fragments, and peak intensity were extracted using Xcalibur (Thermo Fisher Scientific, USA). The local database of authentic standards was obtained from mzVault based on the information of the raw files. Raw metabolome data pretreatment, including peak alignment, peak extraction, and compound identification, was performed using Xcalibur Compound Discoverer 2. The dominant parameters of the database alignment were as follows: mass tolerance of 5 ppm; threshold of signal–noise ratio 1.5; and precursor selection MS. Retention time and MS2 spectra were used for local database alignment. The MS2 spectrum was also aligned with online mass databases, including the Human Metabolome Database (HMDB) (http://www.hmdb.ca/), Kyoto Encyclopedia of Genes and Genomes (KEGG) (https://www.kegg.jp/), and PlantCyc (https://www.plantcyc.org/).

### RNA Sequencing

Total RNA was isolated using the RNAprep Pure Plant Kit (Tiangen Biotech Co., Ltd., Beijing, China) according to the protocol of the manufacturer. The degradation and purity of the RNA were examined by 1% agarose gel electrophoresis and a NanoPhotometer^®^ spectrophotometer (Implen, Westlake Village, CA, USA). Ribonucleic acid integrity was assessed using the RNA Nano 6000 Assay Kit of the Bioanalyzer 2100 system (Agilent Technologies, Santa Clara, CA, USA). The high-quality RNA samples from the tea plants were prepared using an Illumina TruSeq RNA Sample Prep Kit (Illumina, Inc., San Diego, CA, USA), and cDNA libraries were constructed using an Ultra^TM^ RNA Library Prep Kit for Illumina^®^ (New England Biolabs, Inc., Ipswich, MA, USA). The complementary DNAs were purified using Beckman AMPure XP beads (Beckman Coulter, Brea, CA, USA) and subsequently moved to an Agilent High Sensitivity DNA Kit (Agilent 2100, USA) for the detection of inserted cDNA fragments. Afterward, the cDNA libraries were quantified with a Bio-Rad KIT iQ SYBR Green kit (Bio-Rad CFX 96, Bio-Rad Laboratories, Inc., Hercules, CA, USA), and cDNA libraries were subsequently sequenced using a TruSeq SBS Kit v3 (Illumina HiSeq2500, USA). The clean reads were subsequently aligned to the reference genome (http://pcsb.ahau.edu.cn:8080/CSS/). An index of the reference genome was built using HISAT2 v2.0.5, and paired-end clean reads were aligned to the reference genome. A database of splice junctions was generated using HISAT2 based on gene model annotation for an optimized mapping result. Then, the expected number of fragments per kilobase of transcript sequence per million base pairs of each gene was calculated based on the length of the gene and the reads count mapped to the corresponding gene.

### Quantitative Reverse-Transcription PCR Analyses

Nine genes responsible for flavonoids were randomly selected for the validation of gene expression using a quantitative reverse-transcription PCR (qRT-PCR). This information has been described in our previous study including 12 genes (Jiang et al., 2021). The qRT-PCR reactions were conducted using the following parameters: 95°C for 10 min, 45 cycles at 94°C for 10 s, and 58°C for 15 s. Three independent biological replicates and three technical replicates of each reaction were performed using glyceraldehyde 3-phosphate dehydrogenase (GAPDH) as a reference gene. Fluorescence intensity was measured using a LightCycler 480 machine (Roche, Sussex, UK), and the relative expression values of genes were subsequently calculated using the 2^−ΔΔCt^ method.

### Data Analysis

Principal component analysis (PCA) based on metabolites was conducted and visualized using the package ggplot2. The samples were divided into several groups based on the PCA. Fold changes in the relative content of metabolites were calculated according to the formula: variability = maximum/minimum. The metabolites in every two groups with a fold change >2 and a false discovery rate (FDR) <0.05, using the DESeq package, were regarded as different accumulated metabolites (DAMs). Volcano plots were plotted using the package ggplot2. Pearson's correlation coefficient (PCC) values of metabolite–metabolite pairs, gene–gene pairs, or metabolite–gene pairs were calculated using R 3.6.3. The ranks for every pair were obtained based on PCC values from high to low. Then, the mutual rank (MR) was calculated using the formula:$\sqrt{rank\left(AB\right)\times rank\left(BA\right)}$. Mutual rank was converted into network edge weight using the decay function *e*^−(*MR*−1)/100^ ≥ 0.01. The compounds were selected based on an FDR < 0.05 and PCC > 0.8 to construct metabolite correlation networks. The clean reads were aligned to the genome of ‘Shuchazao’ (Wei et al., 2018; Xia et al., 2020) and ‘Longjing 43’ (Wang P. et al., 2020) and then annotated for flavonoid, theanine, and caffeine metabolisms using the Basic Local Alignment Search Tool (National Library of Medicine, USA). The compounds and genes were selected based on an FDR < 0.05 and PCC > 0.4 to construct the gene–metabolite correlation networks. The network was visualized using the Cytoscape software (1991, 1999 Free Software Foundation, Inc.).

## Results

### Overview of Metabolites in Fresh Tea Shoots

**Figure 1:**
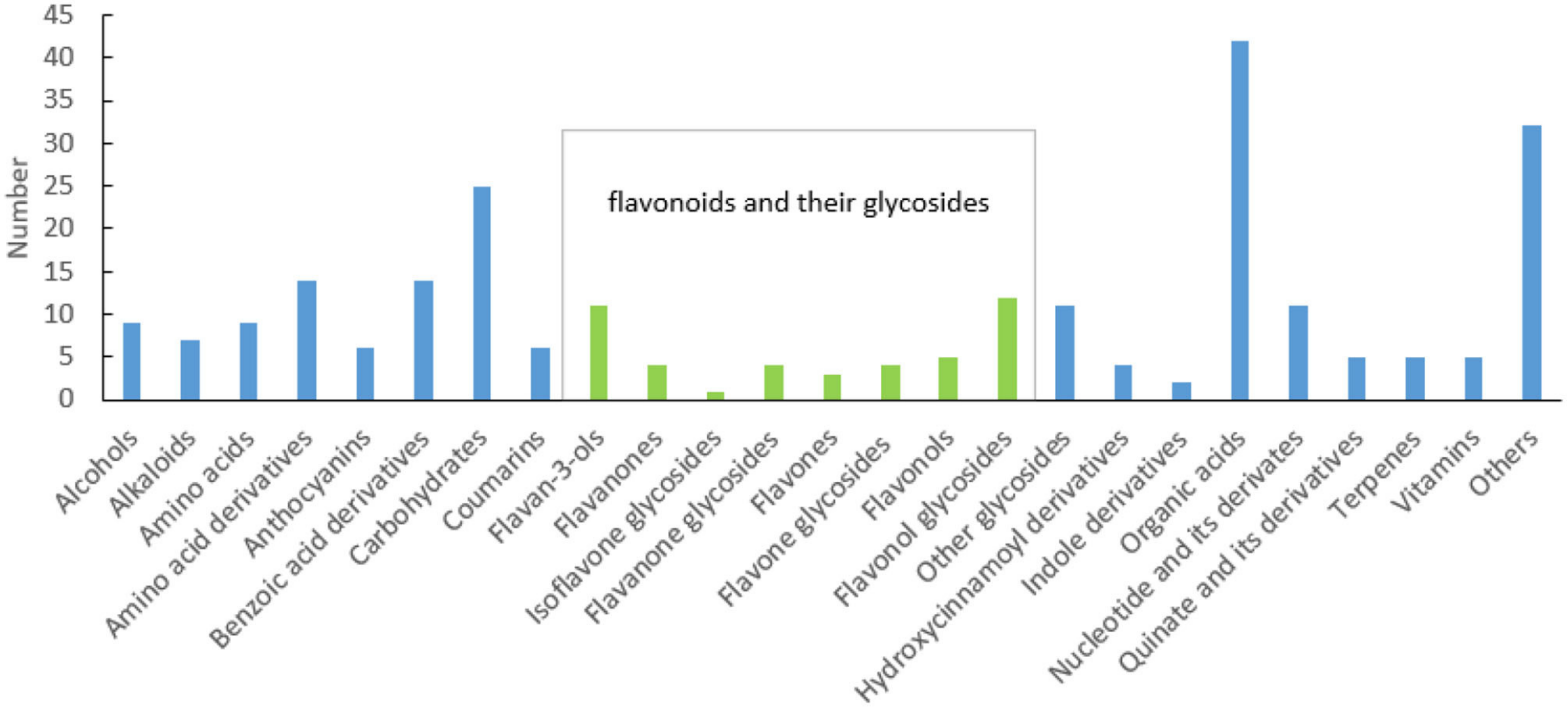
Classification of metabolites in fresh tea shoots.

In the process of sample detection, QC samples were interred every 10 injections to monitor the stability of the detection system. The Pearson correlation coefficient between every two QC samples ranged from 0.99 to 1 (Supplementary Figure 1), indicating that the system was stable. A total of 3,775 features were detected using UPLC-Q-Orbitrap, 251 of which were identified (Supplementary Table 2). Among them, 84 (aa001–aa084) were identified by aligning to the standards, 13 (bb001–bb013) were identified according to the structural information of scientific articles, and the rest (cc001–cc154) were identified through alignment to the public databases. These metabolites can be classified into 25 categories. As described in Figure 1, flavonoids and their glycosides, organic acids, and carbohydrates are the top three classifications. Anthocyanins, flavan-3-ols, flavanones, isoflavone glycosides, flavanone glycosides, flavones, flavone glycosides, flavonols, flavonol glycosides, quinate and its derivatives, and some benzoic acid derivatives are polyphenols. A total of 61 polyphenols were identified. This suggested that there are various polyphenols present in fresh tea shoots. Among them, 29 flavonoid polymers, including flavonoids, sugars, and gallic acids, were identified in fresh tea shoots (Supplementary Table 2). Most of these compounds are catechin polymers and flavonoid glycosides. Procyanidins, ubiquitous and widely secondary metabolites in plants, are condensed flavonoid forms with more than two units of flavanols, corresponding to brown or non-visible-colored pigments (Saigo et al., 2020). Procyanidin B1, procyanidin B3, procyanidin B4, procyanidin C1, and GC-GCG are dimers of catechins, suggesting that a large part of procyanidin is a dimer of catechins. Moreover, glucose and rhamnose are the main glycosides that combine with flavonoids. In addition to the abovementioned compounds, metabolites with galactosylation, rutinosylation, and primeverosylation were also detected in processed tea (Dai et al., 2016). Solubility, molecular stability, and subcellular transport ability increased after glycosylation. Furthermore, flavonols were the majority of aglycones, whose glycosides were glucose and rhamnose, among others. These flavonols were luteolin, myricetin, quercetin, kaempferol, and taxifolin.

**Figure 2:**
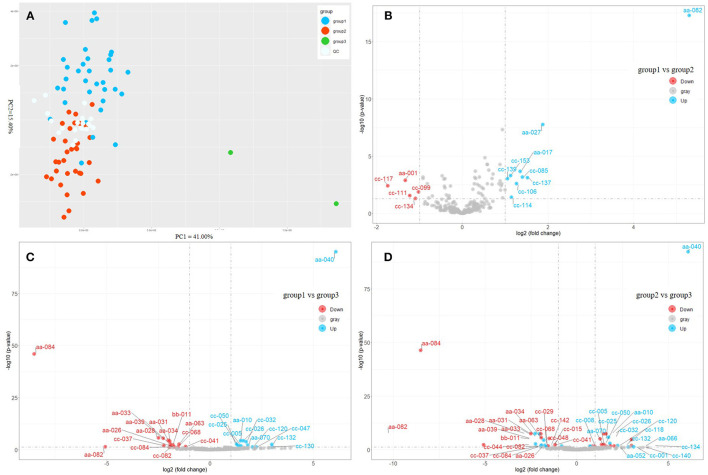
Comparison of metabolic profiles of different tea accessions. **(A)** Principal component analysis of different tea accessions based on the content of metabolites. **(B)** Volcano plot of differentially accumulated metabolites (DAMs) between Group 1 and Group 2. **(C)** Volcano plot of DAMs between Group 1 and Group 3. **(D)** Volcano plot of DAMs between Group 2 and Group 3. aa-001, L-Arginine; aa-010, L-Theanine; aa-017, Nicotinamide; aa-026, Gallocatechin; aa-027, Neochlorogenic acid; aa-028, Theobromine; aa-031, 3,4-Dihydroxybenzaldehyde; aa-033, Procyanidin B3; aa-034, Catechin; aa-039, 4-Hydroxybenzaldehyde; aa-040, Caffeine; aa-063, Procyanidin B1; aa-066, Vitexin-2”-O-rhamnoside; aa-070, Quercitrin; aa082, 1,2-di-O-galloyl-hhdp-glucose; aa-084, GC-GCG; bb-011, diGC-GA; cc-001, Agmatine; cc-005, 4-Amino-4-deoxypentopyranose; cc-015, 2-Acetamido-2-deoxyglucose; cc-025, 4-piperidinecarboxamide; cc-026, Ethanal tetramer; aa-052, Quercetin 3-O-alpha-D-xylopyranoside; cc-008, (2S,4S)-4-Amino-2-hydroxy-2-methylpentanedioic acid; cc-047, N-phenyl-beta-D-glucopyranosylamine; cc-050, Tert-butyl 3-amino-1-methyl-2,3-dioxopropylcarbamate; cc-085, Benzyl 6-O-beta-D-xylopyranosyl-beta-d-glucopyranoside; cc-099, 4,7,8-trihydroxy-3-(4-hydroxyphenyl)dibenzo[b,d]furan-1,2-diyl diacetate ; cc-114, Gallocatechin-(4alpha->8)-epigallocatechin; cc-142, 1,4-anhydro-6-O-dodecanoyl-2,3-bis-O-(2-hydroxyethyl)-D-glucitol; cc-029, 5'-Xanthylic acid; cc-032, Streptamine 4-phosphate; cc-037, Gly-lys; cc-041, 2-Methylcitric acid; cc-044, Hymexazol O-glucoside; cc-048, N-acetyl-l-2-aminoadipic acid; cc-068, Nifurquinazol; cc-082, Aspirin; cc-084, 1-O-vanilloyl-beta-D-glucose; cc-106, Mascaroside; cc-111, Astilbin; cc-117, Tiliroside; cc-118, Olsalazine; cc-120, Cynarine; cc-130, Silandrin; cc-132, Kolaflavanone; cc-134, Lamifiban; cc-137, Methyl 3,4,5-trimethoxycinnamate; cc-139, Propofol; cc-140, 7-Dehydrocholesterol benzoate; cc-153, Beta-D-ethyl glucuronide.

Afterward, we applied the unsupervised PCA to all samples (Figure 2A). The QC samples were distributed at the center of the score graph. In addition to the QC samples, the samples were divided into three groups (Supplementary Table 1). PC1 and PC2 accounted for 41 and 15.4 % of the variation rate, respectively. DMS (different metabolites) [(FDR < 0.05) and |log2(fold change)| > 1] between every two groups were investigated (Figures 2B–D). The contents of nicotinamide, neochlorogenic acid, mascaroside, and propofol were higher in group 1 than those in group 2 (Figure 2B). The contents of l-theanine, caffeine, quercitrin, cynarine, silandrin, and kolaflavanone were higher in group 1 than those in group 3 (Figure 2C). *C. sinensis* var. *sinensis, C. sinensis* var. *assamica*, and *C. sinensis* var. *pubilimba* were distributed in groups 1 and 2. *C. taliensis* and *C. tachangensis* were present in the first group. Group 3 included two tea resources: ‘Jinping 1’, *C. sinensis* var. *assamica*, and ‘Kekecha’ belong to *C. sinensis* var. *pubilimba*, indicating that they were unique. Furthermore, the contents of gallocatechin, theobromine, 3,4-dihydroxybenzaldehyde, procyanidin B1, GC-GCG, diGC-GA, gly-lys, 2-methylcitric acid, nifurquinazo, aspirin, 1-*O*-vanilloyl-β-d-glucose, and cynarine were higher in group 3 than those in the other two groups. The contents of theobromine and GC-GCG were much higher in ‘Kekecha’ than in the other accessions. The common DAM in three pairs, namely, 1,2-Di-*O*-galloyl-HHDP-glucose, was significantly higher in group 3 than in the other two groups. Yang et al. (2008) believed that 1,2-di-*O*-galloyl-HHDP-glucose only exists in *C. taliensis*. However, it has also been observed in *C. sinensis* and *C. tachangensis*. Interestingly, the relative content of 1,2-di-*O*-galloyl-HHDP-glucose was much higher (45.32 times) in ‘Kekecha’, which belongs to *C. sinensis* var. *pubilimba*, compared with *C. taliensis*. Accordingly, 1,2-di-*O*-galloyl-HHDP-glucose was not unique to *C. taliensis*.

### Variability of Metabolites

**Figure 3:**
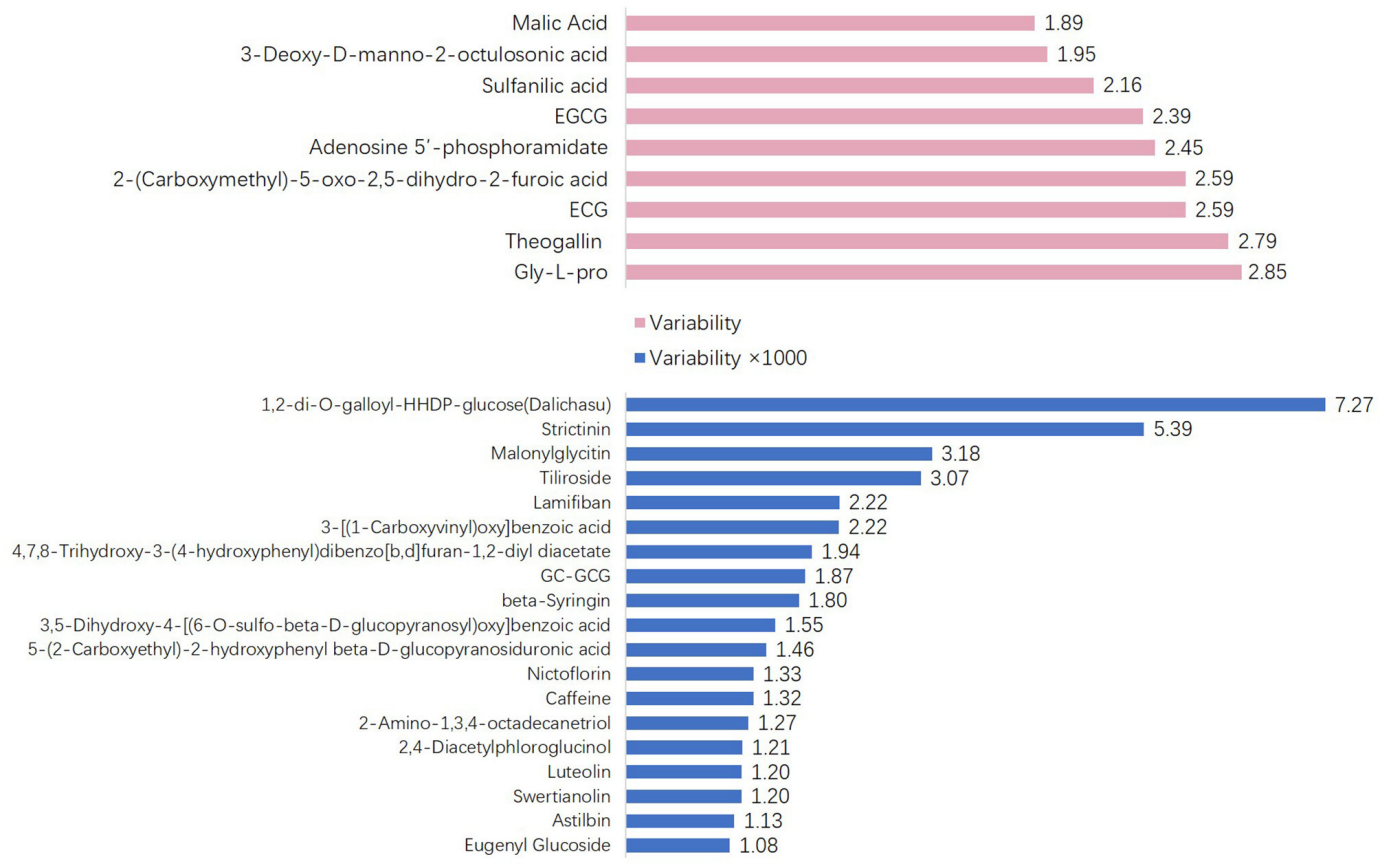
The metabolites whose variability was >5- or more than 1,000-fold.

To explore the variability of metabolites in the fresh shoots of 69 tea accessions, the fold changes in the relative contents of metabolites were calculated according to the formula: variability = maximum/minimum. As shown in Figure 3, the variability of the nine metabolites was less than three. The metabolites with low variability include four organic acids, two catechins, one amino acid derivative, one glycoside, and one nucleotide. These metabolites were conserved, indicating that they play important roles during the normal growth and development of tea plants. In addition to epigallocatechin-3-gallate (EGCG) and gallocatechin-3-gallate (ECG), the remaining were primary metabolites. The content and proportion of catechins are indices for processing suitability. Interestingly, the contents of EGCG and ECG showed low variation among the different varieties. Thus, the variability of other catechins and their derivatives was explored, as presented in Table 1. The variability of catechin-3-gallat (CG), epicatechin (EC), epigallocatechin (EGC), gallocatechin (GC), and C ranged from 5.73 to 16.84, demonstrating their moderate variation, while the variability of the downstream metabolites of catechins [GC-EGC, EGCG3”Me, EGCG4”Me, GC-diGA, GC- gallocatechin-3-gallate (GCG)] was high, ranging between 320.52 and 1865.35. These results suggest that most catechins have significant variations in tea plants, especially catechin polymers. The variability in the 19 cases was more than 1,000 (Figure 3). Dalichasu, strictinin, malonylglycitin, and tiliroside were the top four compounds with a variability of more than 3,000. The first two are alkaloids, and the rest are flavonoid glycosides. This implied that secondary metabolites, especially the downstream metabolites, were more influenced by genetic background.

**Table 1:** Variability of catechins and their derivatives.

**Name**	**Variability folds**
EGCG	2.39
ECG	2.59
Procyanidin B4	4.46
CG	5.73
EC	7.66
Procyanidin C1	7.71
Procyanidin B1	10.92
EGC	11.20
GC	14.63
C	16.84
Procyanidin B3	24.00
GC-EGC	320.52
EGCG3”Me	366.68
EGCG4"Me	800.58
GC-diGA	834.36
GC-GCG	1865.35

### Correlation Among Important Metabolites

**Figure 4:**
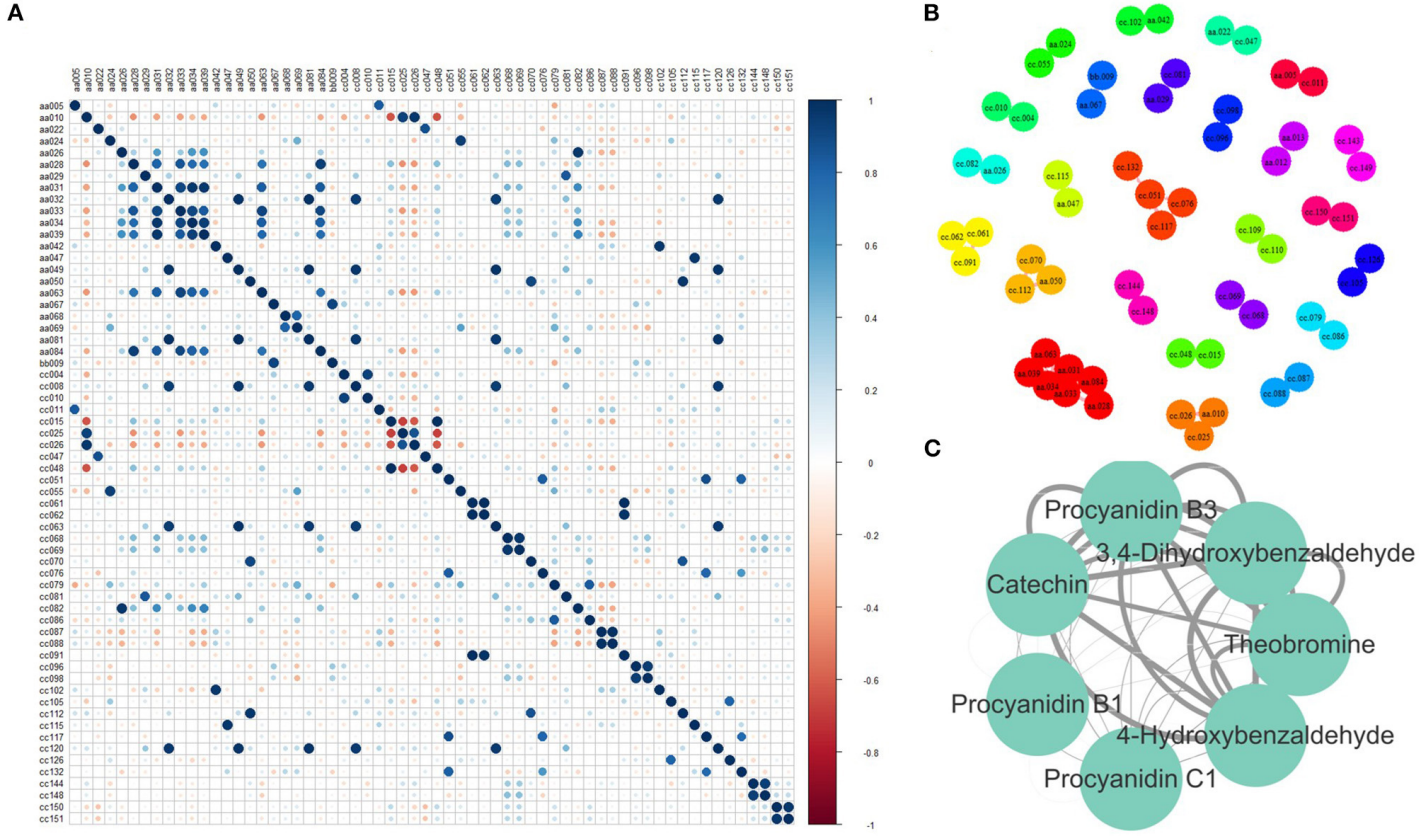
Correlation between metabolites. **(A)** Pearson correlation coefficient (PCC) between every two metabolites. **(B)** Metabolite pairs with a PCC > 0.8. **(C)** Metabolite pairs with a PCC > 0.9 (The width of the edge represents the absolute value of PCC). aa005, Sucrose; aa010, L-Theanine; aa022, Gallic acid; aa024, Theogallin; aa026, GC; aa028, Theobromine; aa029, Methyl gallate; aa031, 3, 4-Dihydroxybenzaldehyde; aa032, 1, 3, 7-Trimethyluric acid; aa033, Procyanidin B3; aa034, C; aa039, 4-Hydroxybenzaldehyde; aa042, TriGA-glucose; aa047, Isoquercetin; aa049, Kaempferitrin; aa050, EGCG4”Me; aa063, Procyanidin B1; aa067, Vitexin; aa068, ECG; aa069, CG; aa081, Theacrine; aa084, GC-GCG; bb009, Eriodictyol C-hexoside; cc010, Glycerophosphoglycerol; cc015, 2-Acetamido-2-deoxyglucose; cc025, 4-Piperidinecarboxamide; cc026, Ethanal tetramer; cc047, N-Phenyl-beta-D-glucopyranosylamine; cc004, 1-Deoxy-D-altro-heptulose 7-phosphate; cc008, (2S, 4S)-4-Amino-2-hydroxy-2-methylpentanedioic acid; cc011, 7-Hydroxy-6-methyl-8-(1-D-ribityl)lumazine; cc051, 2-(3, 4, 5-Trihydroxyphenyl)-3, 4, 5, 7-chromanetetrol; cc070, 5-(2-Carboxyethyl)-2-hydroxyphenyl beta-D-glucopyranosiduronic acid; cc081, 5-Hydroxy-6-methoxy-3-(4-methoxyphenyl)-4-oxo-4H-chromen-7-yl beta-D-glucopyranosiduronic acid; cc144, 2-Linoleoyl-sn-glycero-3-phosphoethanolamine; cc148, 1-Linoleoyl-sn-glycero-3-phosphocholine; cc048, N-Acetyl-L-2-aminoadipic acid; cc055, Adenosine 5′-phosphoramidate; cc061, 3-[(1-Carboxyvinyl)oxy]benzoic acid; cc062, Malonylglycitin; cc063, Histidylglycine; cc068, Nifurquinazol; cc069, beta-D-glucose pentaacetate; cc076, (-)-L-Chicoric acid; cc079, Phenylacetaldehyde; cc082, Aspirin; cc086, 1, 3-Propane sultone; cc087, 7-Ethoxycoumarin; cc088, Sinapaldehyde glucoside; cc091, 2, 4-Diacetylphloroglucinol; cc096, Gentiopicrin; cc098, Sweroside; cc102, 1, 6-bis-O-galloyl-beta-D-glucose; cc105, 4-Carboxy nevirapine; cc112, Malonylgenistin; cc115, N-Adenylylanthranilic acid; cc117, Tiliroside; cc120, Cynarine; cc126, Picrotin; cc132, Kolaflavanone; cc150, Allylcyclohexane; cc151, p-Menth-3-ene.

The correlation among metabolites could present the associations between metabolic content, which helps improve metabolic networks and discover new metabolic pathways. Therefore, PCC was calculated between every two relative contents of the identified metabolites. A total of 49 pairs of metabolites were highly correlated (Figure 4A), as their absolute PCC values were >0.8. Moreover, all the correlations were positive. Among them, the PCC of 29 pairs was >0.9 (Figure 4B). Seven metabolites in one network were further discussed (Figure 4C). They were procyanidin B1, procyanidin B2, procyanidin C1, catechin, 4-hydroxybenzaldehyde, 3,4-dihydroxybenzaldehyde, and theobromine. Procyanidin B1, procyanidin B2, procyanidin C1, and catechin are involved in the flavonoid biosynthesis pathway. 4-hydroxybenzaldehyde, 3,4-dihydroxybenzaldehyde, and theobromine were involved in the biosynthesis of alkaloids derived from the shikimate and caffeine metabolism pathways. Tea plants with high flavonoid content also have high caffeine content, indicating that flavonoids may be closely related to caffeine metabolism.

### Network of Metabolites and Genes Responsible for Flavonoids, Theanine, and Caffeine

**Figure 5:**
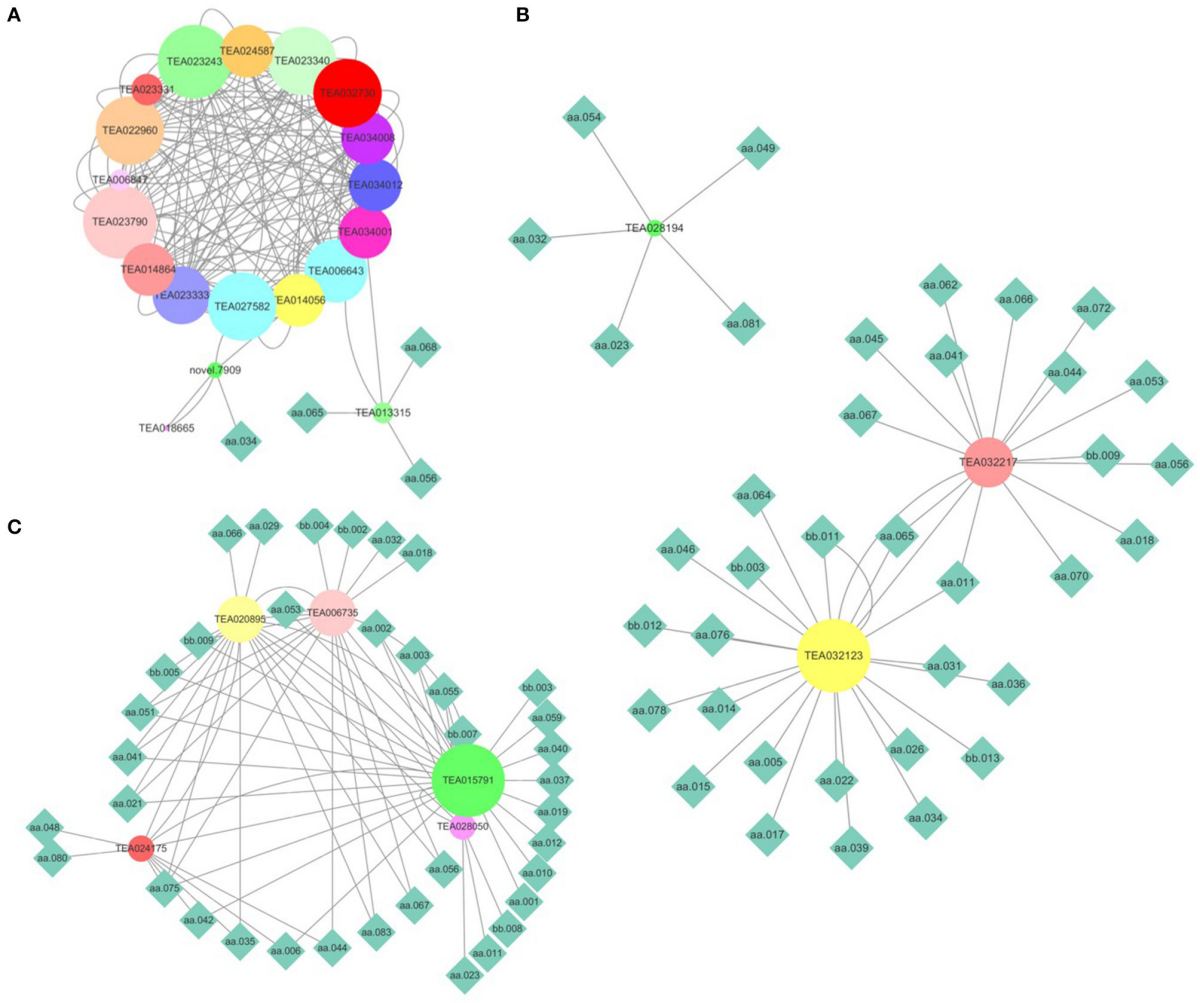
Combined analysis of gene expression levels and metabolite contents. **(A)** Flavonoid metabolism; **(B)** theanine metabolism; **(C)** caffeine metabolism. (The node size reflects the degree value. Nodes were colored based on the hub-gene group). aa001, L-Arginine; aa002, L-Alanine; aa003, L-Aspartic acid; aa005, Sucrose; aa006, L-Proline; aa010, L-Theanine; aa011, Malic Acid; aa012, L-Isoleucine; aa014, Citric acid; aa015, Adenine; aa017, Nicotinamide; aa018, GA-glucose; aa019, L-Phenylalanine; aa021, EGCG3“Me; aa023, Pantothenic acid; aa026, GC; aa029, Methyl gallate; aa031, 3, 4-Dihydroxybenzaldehyde; aa032, 1, 3, 7-Trimethyluric acid; aa034, C; aa035, Strictinin; aa036, Chlorogenic acid; aa037, Procyanidin B4; aa039, 4-Hydroxybenzaldehyde; aa040, Caffeine; aa041, EGCG; aa042, TriGA-glucose; aa044, Schaftoside; aa045, Myricetin 3-O-galactoside; aa046, Rutin; aa048, Ellagic acid; aa049, Kaempferitrin; aa051, Naringin; aa053, Prunin; aa054, Cynaroside; aa055, 2-Hydroxy cinnamic acid; aa056, Myricetin; aa059, Baicalin; aa062, Kaempferol; aa064, Procyanidin C1; aa065, Dihydromyricetin; aa066, Vitexin-2”-O-rhamnoside; aa067, Vitexin; aa068, Epicatechin gallate (ECG); aa070, Quercitrin; aa075, L-Glutathione oxidized; aa076, Guanine; aa078, Esculin; aa080, Shikimic acid; aa081, Theacrine; aa083, GC-diGA; bb002, Glutaric acid; bb003, 2, 5-Dihydroxy benzoic acid O-hexside; bb004, Ellagic acid glucoside; bb005, Protocatechuic acid O-glucoside; bb007, diGA-glucose; bb008, 3-O-p-Coumaroyl quinic acid; bb009, Eriodictyol C-hexoside; bb011, diGC-GA; bb012, Nictoflorin; bb013, 4', 7-Dihydroxyflavanone.

To validate the gene expression level calculated using transcriptomic data, nine genes responsible for flavonoid biosynthesis were randomly selected for a qRT-PCR. The results of the qRT-PCR are consistent with those of RNA sequencing (Jiang et al., 2021). Metabolites are the comprehensive outcomes of gene expression regulated by internal and external factors. A combined analysis of gene expression level and metabolite content was performed to elucidate the transcriptional regulation mechanism underlying the flavonoid, theanine, and caffeine metabolisms. Firstly, the genes in the flavonoid, theanine, and caffeine biosynthesis pathways were screened after being aligned to the reference genomes of ‘Shuchazao’ and ‘Longjing43’. Afterward, PCC was calculated between the gene expression levels and metabolite contents. Finally, the gene-metabolite network was visualized (PCC > 0.4, FDR < 0.05). As shown in Figure 5A, 22 genes play a role in flavonoid metabolism. The genes TEA013315 and novel 0.7909 were highly correlated with epicatechin gallate (aa068), myricetin (aa056), dihydromyricetin (aa065), and catechin (aa034). As presented in Figure 5B, three genes could be divided into three groups based on their regulated metabolites. Among them, TEA28914 was isolated from the center of pantothenic acid (aa023), 1,3,7-trimethyluric acid (aa032), kaempferitrin (aa049), cymaroside (aa054), and theacrine (aa081). The genes TEA032217 and TEA032123 were linked to malic acid (aa011) and dihydromyricetin (aa065). Compared with the genes regulating the flavonoid and theanine metabolisms, five genes in the caffeine biosynthesis pathway were distributed in one group (Figure 5C). In addition, TEA015791 contained 25 metabolites, suggesting its essential role in caffeine metabolism, which deserves further investigation.

## Discussion

The samples from the 68 accessions of tea plants were divided into three groups based on the composition and content of the metabolites (Supplementary Table 1). According to the results of the compound identification, there were abundant tea polyphenols in fresh tea leaves (Figure 1), as the essential signature of tea, both for its higher amount and wider variation compared with other plants. Commercial tea is also rich in various polyphenols, including esterified and non-esterified flavan-3-ols, flavonols, flavone glycosides, phenolic acid ester derivatives, proanthocyanidins, and hydrolysable tannins (Zhuang et al., 2020). Therefore, it was demonstrated that tea polyphenols undergo a series of complex transformations in the manufacturing process. All tea plants were divided into three groups based on the PCA (Figure 2A). *C. taliensis* and *C. tachangensis*, which are important wild relatives of cultivated tea, only existed in the first group. *C. sinensis* var. *sinensis, C. sinensis* var. *assamica*, and *C. sinensis* var. *pubilimba* were distributed in the first and second groups. *C. taliensis* can grow on Mengku Snow Mountain, China, at an altitude of 2,750 m, implying its strong stress resistance (Zhang et al., 2015). Thus, it may harbor abundant gene resources that have strong cold resistance, which can enhance the genetic improvement of cultivated tea. The molecular phylogenetic tree showed that *C. tachangensis, C. gymnogyna*, and *C. taliensis* were clustered into a group based on chloroplast genomes. Additionally, *C. sinensis* var. *sinensis, C. sinensis* var. *assamica*, and *C. sinensis* var. *pubilimba* were clustered into another group (Hao et al., 2019). This result was partially in accordance with chloroplast research. In this study, some *C. sinensis* tea plants were also included in the first group. This suggests that the cultivated tea plants in the first group were closer to the wild ones than those in the second group. ‘Kekecha’, also named cocoa tea, is a natural, low-caffeine, theobromine-rich tea plant that was discovered by Professor Chang Hung-ta in the 1980s (Chang et al., 1988). The variability of 19 metabolites in different tea resources was more than 1,000-fold. Dalichasu, strictinin, malonylglycitin, and tiliroside were the top four compounds, with a variability of more than 3,000-fold. Dalichasu is a polyphenol that is the signature component of *C. taliensis* (Yang et al., 2008). Strictinin was responsible for the anti-influenza activity of Yunnan bitter tea (Kucha), traditionally used for the treatment of the common cold (Lin et al., 2020). Malonylglycitin and tiliroside are isoflavone glycosides and flavonol glycosides, respectively. These compounds were dramatically altered in different tea resources, indicating their peculiar character for distinguishing accessions. Most of the compounds with relatively low variability were the primary metabolites. The conservatism of primary metabolite content is significant for maintaining a stable physiological status. In general, the variation of secondary metabolites was greater than the primary metabolites, demonstrating that the former was more characteristic in different tea accessions. Catechins and their derivatives have high variability as essential secondary metabolites in tea plants. Catechins in tea leaves include four free types, including C, EC, GC, and EGC, and four gallate types, including CG, ECG, GCG, and EGCG. In this study, GCG was not determined because its content was lower than the detection limit. The polymers of GCG and GC-GCG were detected instead. The polymeric forms of flavonoids occur naturally in plants and primarily consist of oligomeric and dimeric catechins. Condensed catechins are proanthocyanidins (Malgorzata and Anna, 2019). Polymerization stabilized flavonoids and caused changes in their specific properties. A polymeric complex catechin compound showed better thermal stability than catechin (Malgorzata and Anna, 2020).

In addition to the physiological functions of tea plants, catechins and their derivatives contribute to the medicinal properties and sensation characteristics of tea infusions for humans. The effect of ‘Benifuki’ tea on human hypertension is mainly the result of the strong inhibitory effect of EGCG3”Me on angiotensin I-converting enzyme activity (Kurita et al., 2010). The effects of EGCG3”Me and EGCG4”Me on reducing blood pressure, anti-anaphylaxis, and anti-inflammation were more significant than those of EGCG. Catechins and epicatechins are absorbed from the human intestinal tract after transforming into *O*-methylated derivatives (Suzuki et al., 2000). Therefore, methylated catechins are more easily absorbed than catechins. Caffeoyl-CoA 3-*O-*methyltransferase (CCoAOMT) has been reported to directly catalyze the synthesis of EGCG3′′Me. On the other hand, *CsbHLH62, CsWRKY31*, and *CsWRKY48* might negatively regulate the biosynthesis of EGCG3”Me by trans-repressing its expression of the downstream genes *CCoAOMT, CsLAR*, and *CsDFR* of the EGCG3”Me biosynthetic pathway (Luo et al., 2018, 2019). A tandem mass spectrum analysis characterized EGCG4”Me as resulting from epicatechin carbocation and the methyl group of methanol (Wang X. et al., 2020). Most catechin polymers are GC derivatives. Our previous research suggested that GC accounts for a small amount of the total catechin content (Jiang et al., 2020). It is likely that GC formed polymers with EGC, GCG, and gallic acid (GA) as one of the factors for its low absolute content in tea leaves. In this study, the contents of GC-GCG and theobromine were extremely higher in ‘Kekecha’ than in other accessions, which is consistent with the results of previous studies (Peng et al., 2019). The monomeric units of procyanidins were linked through a C4–C8 or C4–C6 bond (B-type), which can coexist with an additional C2–O–C7 or less abundant C2–O–C5 bond (A-type). Procyanidins were detected and were all B-type (procyanidins B1, B3, and B4) (Table 1). It was also revealed that the bonds of catechin C1 are most commonly C-4 to C-8 (Li et al., 2013).

In addition to polymerization, glycosylation is one of the most popular methods for improving stability using various enzymes that possess transglycosylation activity (Cho et al., 2011). Phenolic acid, flavonol, flavone, and isoflavone are catalyzed into glycosylated compounds by the flavonoid UDP-glycosyltransferase, which transfers glycosyl groups from activated glycosyl donors to flavonoids (Achnine et al., 2005). In green and black tea, flavonol glycosides cover subclasses of quercetin *O-*glycosides, kaempferol *O-*glycosides, and myricetin *O-*glycosides (Li et al., 2020). In Pu'er tea, quercetin, myricetin, and kaempferol are the main aglycones of tea flavonols (Jiang et al., 2018). This indicated that these flavonol glycosides are the flavor components of tea and the substances possessing anti-stress functions in fresh tea leaves. Glycosides, an important substrate of glucosidase during tea fermentation, contribute to the nutrition and flavor of tea. The glycosylation of flavonol aglycones is the last step in flavonol biosynthesis, catalyzed by uridine diphospho-glycosyltransferases (Xie et al., 2020). The main factors that lead to low or no detection of glycosides were as follows: (1) β-primeverosides were the aroma precursors in fresh tea leaves. The content of glycosides in fresh tea shoots was relatively lower than that in processed tea, as a number of these compounds are formed during tea processing (Zhou et al., 2017). (2) We did not use a specific method to explore glycosylated compounds. Presently, a very small portion of MS features can be structurally annotated (Da Silva et al., 2015; Nash and Dunn, 2019). For instance, only 6.65% of the features were identified in this study. From the deamination and decarboxylation reaction of theanine, *N-*ethyl-pyrrolidone may combine with C6 or C8 of the A ring of catechins to produce a catechin–theanine complex (Li et al., 2018). In addition, catechin–theanine complexes were not detected in this study, wherein their presence at levels below the detection limit is one possible reason.

To investigate the gene regulatory network of flavonoids, theanine, and caffeine syntheses in tea plants, a gene–metabolite network was constructed according to the PCC value. Only four metabolites were closely associated with the 22 genes responsible for flavonoid biosynthesis. The relationship between genes was closer than that between genes and metabolites. In contrast, more metabolites, rather than genes, were in the network of theanine and caffeine. Flavonoid biosynthesis may be regulated in a more complicated manner than theanine or caffeine biosynthesis. According to current knowledge, there are 12 structural genes (excluding alleles) in the flavonoid metabolism pathway. Five and four structural genes (excluding alleles) are in the theanine and caffeine metabolism pathways, respectively (Wei et al., 2018; Zhu et al., 2019). Theacrine, 1,3,7,9-tetramethyluric acid, showed an effect similar to that of caffeine that enhanced locomotor activation through dopaminergic and adenosinergic systems while exhibiting superior toxicity to caffeine (Ashburn et al., 2019). It is the major purine alkaloid in the leaves of a special Chinese tea, *C. assamica* var. *kucha* Hung T. Chang and H.S.Wang, a variety of *Theaceae* (Zheng et al., 2002). Morphologically, *kucha* is not significantly different from *sinensis*, so it is usually classified as *C. sinensis*. It was also the dominant purine alkaloid detected in *C. sinensis* var. *pusanensis* Kurihara (Li et al., 2017). However, theacrine content was not related to tea varieties. More interestingly, it was highly correlated with the theanine metabolism, similar to the gene–metabolite (theanine) network. The network of metabolites and genes revealed that candidate genes regulated the related metabolites.

## Conclusions and Future Prospects

The non-volatile metabolic profiling of fresh shoots from 68 tea accessions was performed based on the results of non-targeted metabolomics. First, 251 metabolites were identified by aligning to the standards, structural information of scientific articles, or public databases. All tea samples could be classified into three groups. Varieties of *C. sinensis*, involving var. *sinensis*, var. *assamica*, and var. *pubilimba*, were distributed in the first and second groups. The wild tea plants, including *C. taliensis* and *C. tachangensis*, only existed in the first group. ‘Jinping 1’ belonged to *C. sinensis* var. *assamica* and ‘Kekecha’ belonged to *C. sinensis* var. *pubilimba* in the third group. Second, the variabilities of the metabolites were counted. Compared with the primary metabolites, the secondary metabolites, especially the downstream metabolites, were more influenced by genetic background. Third, flavonoid polymers had attracted increasing attention for their importance in tea growth and development. Catechins were commonly formed in their dimers, called procyanidin. Glucose and rhamnose were the main glycosides that combine with flavonoids. Fourth, correlations among metabolites were conducted based on PCC. The contents of flavonoids and caffeine were likely positively correlated in most circumstances. Finally, a network of metabolites and genes responsible for flavonoids, theanine, and caffeine was constructed. Results showed that TEA013315 and novel 0.7909 were highly correlated with epicatechin gallate. The gene TEA28914 was isolated from the center of pantothenic acid, 1,3,7-trimethyluric acid, kaempferitrin, cymaroside, and theacrine. The genes TEA032217 and TEA032123 were linked to malic acid and dihydromyricetin. The regulatory mechanisms underlying these genes and metabolites require further study.

## Data Availability Statement

The data presented in the study are deposited in the NCBI repository, accession number PRJNA760638. The raw data were published on https://www.ncbi.nlm.nih.gov/bioproject/760638.

## Author Contributions

LC, J-QM, and C-KJ designed the experiments. C-KJ, Z-LL, and X-YL conducted the experiments. C-KJ analyzed and wrote the manuscript. LC and SE polished the manuscript. All authors contributed to the article and approved the submitted version.

## Funding

This work was supported by the National Natural Science Foundation of China (32072631, U19A2030), the China Agriculture Research System of MOF and MARA, and the Chinese Academy of Agricultural Sciences through the Agricultural Science and Technology Innovation Program (CAAS-ASTIP-2017-TRICAAS).

## Conflict of Interest

The authors declare that the research was conducted in the absence of any commercial or financial relationships that could be construed as a potential conflict of interest.

## Publisher's Note

All claims expressed in this article are solely those of the authors and do not necessarily represent those of their affiliated organizations, or those of the publisher, the editors and the reviewers. Any product that may be evaluated in this article, or claim that may be made by its manufacturer, is not guaranteed or endorsed by the publisher.

## Abbreviations

C: catechin
EC: epicatechin
GC: gallocatechin
EGC: epigallocatechin
CG: catechin-3-gallate
ECG: epicatechin-3-gallate
GCG: gallocatechin-3-gallate
GA: gallic acid
EGCG: epigallocatechin-3-gallate
QC: quality control
RT: retention time
PCA: principal component analysis
FDR: false discovery rate
DAM: different accumulated metabolite
PCC: Pearson correlation coefficient
MR: mutual rank.
