# Supplementary material for: Non-Volatile Metabolic Profiling and Regulatory Network Analysis in Fresh Shoots of Tea Plant and Its Wild Relatives
Source: Front Plant Sci. 2021 Oct 1;12:746972. doi: 10.3389/fpls.2021.746972 (PMC8519607; doi:10.3389/fpls.2021.746972)
Supplement: Supplementary file 1 [file Data_Sheet_1.docx]

Supplementary Material

# Supplementary Figures and Tables

## Supplementary Figure


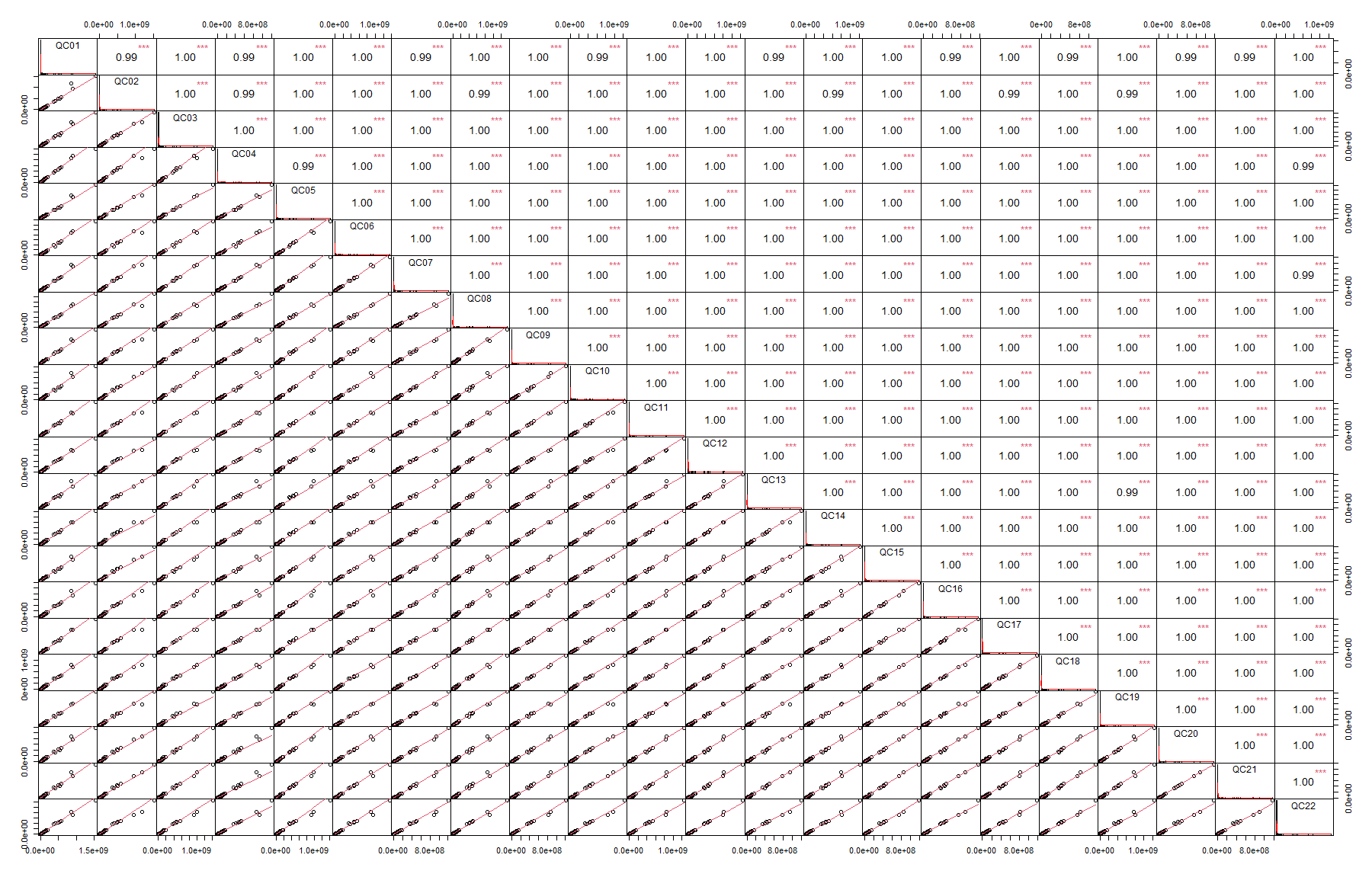


Figure S1 PCC value between QC samples.

## Supplementary Tables

Table S1 Sample information

| **Full name** | **Short name** | **group** | **Species** |
| --- | --- | --- | --- |
| Wushanban qingmingcha | WB | group1 | *Camellia sinensis* (L.) O. Kuntze var. *sinensis* |
| Baiye 1 | B1 | group2 | *C. sinensis* (L.) O. Kuntze var. *sinensis* |
| Baofu 1 | BF | group2 | *C. sinensis* (L.) O. Kuntze var. *sinensis* |
| Luoshe | LS | group2 | *C. sinensis* (L.) O. Kuntze var. *sinensis* |
| Lianyuan qiqu | LQ | group1 | *C. sinensis* (L.) O. Kuntze var. *sinensis* |
| Xiancha | XC | group1 | *C. sinensis* (L.) O. Kuntze var. *sinensis* |
| Lantian | LT-1 | group2 | *C. sinensis* (L.) O. Kuntze var. *sinensis* |
| Yingshuang | YS | group2 | *C. sinensis* (L.) O. Kuntze var. *sinensis* |
| Maolv | ML | group2 | *C. sinensis* (L.) O. Kuntze var. *sinensis* |
| Yinsun | YI | group2 | *C. sinensis* (L.) O. Kuntze var. *sinensis* |
| Baihaozao | BH | group2 | *C. sinensis* (L.) O. Kuntze var. *sinensis* |
| Anhui 3 | AH | group2 | *C. sinensis* (L.) O. Kuntze var. *sinensis* |
| Aidiancha | AD | group1 | *C. sinensis* var. *pubilimba* Chang |
| Guihong 3 | GH | group2 | *C. sinensis* (L.) O. Kuntze var. *sinensis* |
| Lingtou dancong | LC | group1 | *C. sinensis* (L.) O. Kuntze var. *sinensis* |
| Jinping 1 | JP | group3 | *C. sinensis* var. *assamica* (Masters) Kitamura |
| Yongping 4 | Y4 | group1 | *C. sinensis* var. *assamica* (Masters) Kitamura |
| Yuanyang 4 | YY | group1 | *C. sinensis* var. *assamica* (Masters) Kitamura |
| Longchuan 9 | L9 | group1 | *C. sinensis* (L.) O. Kuntze var. *sinensis* |
| Xingyi 5 | X5 | group1 | *C. sinensis* var. *assamica* (Masters) Kitamura |
| Xingyi 4 | X4 | group1 | *C. tachangensis* F. C. Zhang |
| Longjin 43 | LJ | group1 | *C. sinensis* (L.) O. Kuntze var. *sinensis* |
| Longchuan longjinxiaoye | LL | group1 | *C. sinensis* (L.) O. Kuntze var. *sinensis* |
| Langdicha | LD | group1 | *C. sinensis* var. *assamica* (Masters) Kitamura |
| Malipo mada 1 | MM | group1 | *C. sinensis* var. *assamica* (Masters) Kitamura |
| Zhenyuan zhengtaiyecha | ZZ | group1 | *C. taliensis* (W. W. Smith) Melchior |
| Yuncha qirui | YQ | group1 | *C. sinensis* var. *assamica* (Masters) Kitamura |
| Yuncha purui | YP | group1 | *C. sinensis* var. *assamica* (Masters) Kitamura |
| Yunxian huangyehuangya | YH | group2 | *C. sinensis* var. *assamica* (Masters) Kitamura |
| Nanjian 7 | NJ | group1 | *C. sinensis* var. *assamica* (Masters) Kitamura |
| Shuangbai 7 | SB | group1 | *C. sinensis* (L.) O. Kuntze var. *sinensis* |
| Houshancha | HS | group2 | *C. sinensis* (L.) O. Kuntze var. *sinensis* |
| Lechang langtiankucha | LK | group2 | *C. sp.* |
| Beiyue Danzhu | BD | group1 | *C. sinensis* var. *pubilimba* Chang |
| Huangshan kucha | HK | group1 | *C. sinensis* (L.) O. Kuntze var. *sinensis* |
| Asamucha | AS | group1 | *C. sinensis* var. *assamica* (Masters) Kitamura |
| Shanglin 3 | SL | group2 | *C. sinensis* var. *pubilimba* Chang |
| Bobai 1 | BB | group2 | *C. sinensis* var. *pubilimba* Chang |
| Xinjin qunti | XQ | group1 | *C. sinensis* var. *assamica* (Masters) Kitamura |
| Dapo qunti | DQ | group1 | *C. sinensis* var. *assamica* (Masters) Kitamura |
| Wuxi dachahshu | WD | group2 | *C. sinensis* (L.) O. Kuntze var. *sinensis* |
| Maomicha | MC | group2 | *C. sinensis* (L.) O. Kuntze var. *sinensis* |
| Baodian qunti 2 | BQ | group1 | *C. sinensis* var. *assamica* (Masters) Kitamura |
| Zhongjian qunti | ZQ | group1 | *C. sinensis* var. *assamica* (Masters) Kitamura |
| Zhongcha 108 | ZC | group1 | *C. sinensis* (L.) O. Kuntze var. *sinensis* |
| Jinguanyin | JG | group2 | *C. sinensis* (L.) O. Kuntze var. *sinensis* |
| Yuemingxiang | YM | group2 | *C. sinensis* (L.) O. Kuntze var. *sinensis* |
| Shuchazao | SC | group2 | *C. sinensis* (L.) O. Kuntze var. *sinensis* |
| Echa 1 | EC | group2 | *C. sinensis* (L.) O. Kuntze var. *sinensis* |
| Qimen 10 | QM | group2 | *C. sinensis* (L.) O. Kuntze var. *sinensis* |
| Huangjinya | HJ | group1 | *C. sinensis* (L.) O. Kuntze var. *sinensis* |
| Huangmeigui | HM | group2 | *C. sinensis* (L.) O. Kuntze var. *sinensis* |
| Baijiguan | BJ | group1 | *C. sinensis* (L.) O. Kuntze var. *sinensis* |
| Lizhi Wulong | LW | group1 | *C. sinensis* (L.) O. Kuntze var. *sinensis* |
| Tieluohan | TL | group2 | *C. sinensis* (L.) O. Kuntze var. *sinensis* |
| Kugua | KG | group1 | *C. sinensis* (L.) O. Kuntze var. *sinensis* |
| Wuyi baimudan | WY | group2 | *C. sinensis* (L.) O. Kuntze var. *sinensis* |
| Baiqilan | BL | group2 | *C. sinensis* (L.) O. Kuntze var. *sinensis* |
| Rougui | RG | group2 | *C. sinensis* (L.) O. Kuntze var. *sinensis* |
| Lebai 5820 | LB | group2 | *C. sinensis* var. *pubilimba* Chang |
| Luoding hongyazhong | LH | group2 | *C. sinensis* (L.) O. Kuntze var. *sinensis* |
| Dongming 1 | DM | group2 | *C. sinensis* (L.) O. Kuntze var. *sinensis* |
| Qianshantou | QS | group2 | *C. sinensis* var. *assamica* (Masters) Kitamura |
| Longjin ziya | LZ | group2 | *C. sinensis* (L.) O. Kuntze var. *sinensis* |
| Jiaming 1 | JM | group1 | *C. sinensis* (L.) O. Kuntze var. *sinensis* |
| Kekecha | KK | group3 | *C. sinensis* var. *pubilimba* Chang |
| Ziyan | ZY | group2 | *C. sinensis* (L.) O. Kuntze var. *sinensis* |
| Zijuan | ZJ | group1 | *C. sinensis* var. *assamica* (Masters) Kitamura |

S2 Identified compounds

| **Identifier** | **Name** | **Formula** | **Molecular Weight** | **RT [min]** | **Family** | **MS2 Fragments** |
| --- | --- | --- | --- | --- | --- | --- |
| aa001 | L-Arginine | C_6_H_14_N_4_O_2_ | 174.1117 | 0.826 | Amino acids | 70.07, 175.12, 60.06, 116.07, 130.10 |
| aa002 | L-Alanine | C_3_H_7_NO_2_ | 89.04775 | 0.85 | Amino acids | 90.06, 56.97, 50.06, 54.12, 54.67 |
| aa003 | L-Aspartic acid | C_4_H_7_NO_4_ | 133.0375 | 0.853 | Amino acids | 74.02, 88.04, 116.03, 70.03, 134.04 |
| aa004 | L-Glutamic acid | C_5_H_9_NO_4_ | 147.0532 | 0.861 | Amino acids | 84.04, 102.05, 130.05, 85.05 |
| aa005 | Sucrose | C_12_H_22_O_11_ | 342.1167 | 0.891 | Carbohydrates | 59.01, 71.01, 89.02, 101.02, 341.11 |
| aa006 | L-Proline | C_5_H_9_NO_2_ | 115.0634 | 0.906 | Amino acids | 70.07, 116.07, 71.07, 117.07 |
| aa007 | Betaine | C_5_H_11_NO_2_ | 117.079 | 0.913 | Alkaloids | 118.09, 59.07, 58.07, 119.09, 60.08 |
| aa008 | DL-Tartaric acid | C_4_H_6_O_6_ | 150.0165 | 0.913 | Organic acids | 72.99, 87.01, 149.01, 103.00 |
| aa009 | D-Erythrose | C_4_H_8_O_4_ | 120.0424 | 0.922 | Carbohydrates | 71.01, 59.01, 89.02, 117.93 |
| aa010 | L-Theanine | C_7_H_14_N_2_O_3_ | 174.1004 | 0.967 | Amino acids | 173.09, 155.08, 84.05, 74.02, 128.04 |
| aa011 | Malic Acid | C_4_H_6_O_5_ | 134.0216 | 0.982 | Organic acids | 115.00, 71.01, 133.01, 72.99, 89.02 |
| aa012 | L-Isoleucine | C_6_H_13_NO_2_ | 131.0947 | 1.064 | Amino acids | 86.10, 69.07, 87.10, 132.10 |
| aa013 | L-Leucine | C_6_H_13_NO_2_ | 131.0947 | 1.209 | Amino acids | 86.10, 87.10, 132.10 |
| aa014 | Citric acid | C_6_H_8_O_7_ | 192.0271 | 1.218 | Organic acids | 111.01, 87.01, 85.03, 191.02 |
| aa015 | Adenine | C_5_H_5_N_5_ | 135.0546 | 1.235 | Nucleotide and its derivates | 134.05, 107.04, 92.03, 135.05, 108.04 |
| aa016 | Succinic acid | C_4_H_6_O_4_ | 118.0267 | 1.308 | Organic acids | 73.03, 117.02, 99.01, 116.93, 74.03 |
| aa017 | Nicotinamide | C_6_H_6_N_2_O | 122.0481 | 1.341 | Vitamins | 123.06, 80.05, 96.04, 124.06, 81.05 |
| aa018 | GA-glucose | C_13_H_16_O_10_ | 332.0747 | 1.476 | Glycosides | 59.01, 123.01, 331.07, 125.02, 169.01 |
| aa019 | L-Phenylalanine | C_9_H_11_NO_2_ | 165.0791 | 1.557 | Amino acids | 120.08, 103.05, 121.08, 131.05 |
| aa020 | Xanthine | C_5_H_4_N_4_O_2_ | 152.0335 | 1.621 | Nucleotide and its derivates | 72.94, 113.96, 153.04, 90.95, 131.97 |
| aa021 | EGCG3"Me | C_10_H_13_N_5_O_4_ | 267.0967 | 1.689 | Flavan-3-ols | 181.07, 182.08, 138.07, 137.08, 108.06 |
| aa022 | Gallic acid (GA) | C_7_H_6_O_5_ | 170.0216 | 1.916 | Benzoic acid derivatives | 183.01, 119.05, 325.18, 145.03, 184.02 |
| aa023 | Pantothenic acid | C_9_H_17_NO_5_ | 219.1108 | 2.058 | Vitamins | 209.05, 167.03, 123.05, 125.02, 95.05 |
| aa024 | Theogallin (Trihydroxybenzoic acid glycoside) | C_14_H_16_O_10_ | 344.0745 | 2.247 | Quinate and its derivatives | 173.05, 119.05, 93.03, 163.04, 191.06 |
| aa025 | 7-Methylxanthine | C_6_H_6_N_4_O_2_ | 166.0491 | 2.586 | Nucleotide and its derivates | 119.05, 151.04, 185.02, 93.03, 145.03 |
| aa026 | Gallocatechin (GC) | C_15_H_14_O_7_ | 306.0741 | 3.224 | Flavan-3-ols | 78.96, 96.96, 96.97, 153, 409.24 |
| aa027 | Neochlorogenic acid (5-O-Caffeoylquinic acid) | C_16_H_18_O_9_ | 354.0954 | 3.613 | Quinate and its derivatives | 411.27, 412.27, 409.16, 410.17, 413.28 |
| aa028 | Theobromine | C_7_H_8_N_4_O_2_ | 180.0647 | 4.138 | Alkaloids | 255.03, 227.04, 284.03, 417.08, 285.04 |
| aa029 | Methyl gallate | C_8_H_8_O_5_ | 184.0372 | 4.404 | Benzoic acid derivatives | 135.05, 375.07, 191.06, 161.02, 88.99 |
| aa030 | Epigallocatechin (EGC) | C_15_H_14_O_7_ | 306.0741 | 4.405 | Flavan-3-ols | 78.96, 96.97, 80.92, 96.96, 87.01 |
| aa031 | 3,4-Dihydroxybenzaldehyde | C_7_H_6_O_3_ | 138.0316 | 4.405 | Benzoic acid derivatives | 153.02, 425.14, 179.03, 255.06, 331.04 |
| aa032 | 1,3,7-Trimethyluric acid | C_8_H_10_N_4_O_3_ | 210.0754 | 4.832 | Organic acids | 290.08, 91.05, 310.6, 246.01, 74.1 |
| aa033 | Procyanidin B3 | C_30_H_26_O_12_ | 578.1427 | 4.95 | Anthocyanins | 125.02, 273.08, 97.03, 169.01, 255.07 |
| aa034 | (+) Catechin (C) | C_15_H_14_O_6_ | 290.0793 | 4.955 | Flavan-3-ols | 78.96, 88.99, 153, 60.99, 125.02 |
| aa035 | Strictinin | C_27_H_22_O_18_ | 634.0813 | 5.184 | Glycosides | 78.96, 153, 191.06, 119.05, 96.97 |
| aa036 | Chlorogenic acid (3-O-Caffeoylquinic acid) | C_16_H_18_O_9_ | 354.0953 | 5.351 | Quinate and its derivatives | 96.96, 125.02, 391.07, 79.96, 150 |
| aa037 | Procyanidin B4 | C_30_H_26_O_12_ | 578.143 | 5.686 | Anthocyanins | 437.19, 437.23, 438.2, 438.24, 439.2 |
| aa038 | Epicatechin (EC) | C_15_H_14_O_6_ | 290.0793 | 5.855 | Flavan-3-ols | 147.04, 237.03, 131.05, 103.05, 90.98 |
| aa039 | 4-Hydroxybenzaldehyde | C_7_H_6_O_2_ | 122.0364 | 5.855 | Benzoic acid derivatives | 213.03, 125.02, 167.03, 171.02, 195.02 |
| aa040 | Caffeine | C_8_H_10_N_4_O_2_ | 194.0804 | 6.256 | Alkaloids | 125.02, 168.01, 124.02, 289.07, 109.03 |
| aa041 | (-)-Epigallocatechin 3-O-gallate(EGCG) | C_22_H_18_O_11_ | 458.0853 | 6.746 | Flavan-3-ols | 153.02, 467.19, 163.04, 131.05, 153.03 |
| aa042 | TriGA-glucose | C_27_H_24_O_18_ | 636.097 | 7.266 | Benzoic acid derivatives | 151.04, 151, 115, 133.01, 71.01 |
| aa043 | Quercetin | C_15_H_10_O_7_ | 302.0424 | 7.783 | Flavonols | 119.05, 163.04, 125.02, 135.05, 93.03 |
| aa044 | Schaftoside (Apigenin 6-C-arabinoside-8-C-glucoside) | C_26_H_28_O_14_ | 564.1485 | 7.485 | Flavone glycosides | 469.13, 147.04, 119.05, 470.14, 91.05 |
| aa045 | Myricetin 3-O-galactoside | C_21_H_20_O_13_ | 480.0907 | 7.488 | Flavonol glycosides | 167.04, 125.02, 123.01, 173.05, 153.02 |
| aa046 | Rutin | C_27_H_30_O_16_ | 610.1539 | 8.163 | Flavonol glycosides | 279.23, 78.96, 476.28, 280.24, 140.01 |
| aa047 | Isoquercetin | C_21_H_20_O_12_ | 464.0959 | 8.384 | Flavonol glycosides | 139.04, 326.05, 307.08, 363.04, 140.04 |
| aa048 | Ellagic acid | C_14_H_6_O_8_ | 302.0065 | 8.51 | Benzoic acid derivatives | 125.02, 169.01, 211.02, 483.08, 93.03 |
| aa049 | Kaempferitrin (kaempferol 3,7-dirhamnoside) | C_27_H_30_O_14_ | 578.1639 | 8.576 | Flavonol glycosides | 153.02, 163.04, 485.2, 317.08, 135.04 |
| aa050 | EGCG4"Me | C_23_H_20_O_11_ | 472.101 | 8.094 | Flavan-3-ols | 125.02, 169.01, 211.02, 483.08, 93.03 |
| aa051 | Naringin (Naringenin 7-O-neohesperidoside) | C_27_H_32_O_14_ | 580.1801 | 8.636 | Flavanone glycosides | 487.21, 487.08, 488.22, 488.09, 185.04 |
| aa052 | Quercetin 3-O-а-D-xylopyranoside | C_20_H_18_O_11_ | 434.0853 | 8.65 | Flavonol glycosides | 125.02, 169.01, 289.07, 109.03, 441.08 |
| aa053 | Prunin (Naringenin 7-O-glucoside) | C_21_H_22_O_10_ | 434.1218 | 8.685 | Flavanone glycosides | 183.03, 124.02, 168.01, 125.02, 140.01 |
| aa054 | Cynaroside | C_21_H_20_O_11_ | 448.1008 | 8.717 | Flavone glycosides | 125.02, 169.01, 97.03, 273.08, 137.02 |
| aa055 | 2-Hydroxy cinnamic acid | C_9_H_8_O_3_ | 164.0474 | 8.722 | Hydroxycinnamoyl derivatives | 211.02, 168.01, 169.01, 125.02, 505.06 |
| aa056 | Myricetin | C_15_H_10_O_8_ | 318.0379 | 8.929 | Flavonols | 507.07, 337.05, 508.08, 505.23, 506.23 |
| aa057 | Eriodictyol | C_15_H_12_O_6_ | 288.0637 | 8.961 | Flavanones | 125.02, 463.22, 169.01, 71.01, 137.02 |
| aa058 | Kaempferin (Afzelin, Kaempferol 3-rhamnoside) | C_21_H_20_O_10_ | 432.1059 | 8.984 | Flavonol glycosides | 135.05, 255.03, 191.06, 227.03, 173.09 |
| aa059 | Baicalin | C_21_H_18_O_11_ | 446.0856 | 9.015 | Flavone glycosides | 213.03, 171.02, 125.02, 195.02, 108.05 |
| aa060 | Tricetin (5,7,3',4',5'-Pentahydroxyflavone) | C_15_H_10_O_7_ | 302.0429 | 9.054 | Flavones | 184.07, 86.1, 520.34, 125, 104.11 |
| aa061 | Abscisic acid | C_15_H_20_O_4_ | 264.1363 | 9.279 | Organic acids | 331.07, 59.01, 125.02, 169.01, 123.01 |
| aa062 | Kaempferol | C_15_H_10_O_6_ | 286.0481 | 9.648 | Flavonols | 271.03, 300.03, 255.03, 463.09, 125.02 |
| aa063 | Procyanidin B1 | C_30_H_26_O_12_ | 578.1429 | 4.728 | Anthocyanins | 191.06, 85.03, 192.06, 93.03, 255.03 |
| aa064 | Procyanidin C1 | C_30_H_26_O_12_ | 578.143 | 6.964 | Anthocyanins | 131.05, 103.05, 132.05, 104.06, 407.33 |
| aa065 | Dihydromyricetin | C_15_H_12_O_8_ | 320.0535 | 6.226 | Flavonols | 125.02, 169.01, 150.03, 149.02, 80.97 |
| aa066 | Vitexin-2''-o-rhamnoside | C_27_H_30_O_14_ | 578.1638 | 8.168 | Coumarins | 125.02, 167.04, 209.05, 123.05, 239.06 |
| aa067 | Vitexin (Apigenin 8-C-glucoside) | C_21_H_20_O_10_ | 432.1058 | 8.37 | Flavones | 59.01, 71.01, 149.02, 89.02, 329.14 |
| aa068 | Epicatechin gallate (ECG) | C_22_H_18_O_10_ | 442.0904 | 8.232 | Flavan-3-ols | 78.96, 153, 171.01, 245.04, 96.97 |
| aa069 | Epicatechin gallate (CG) | C_22_H_18_O_10_ | 442.0899 | 8.431 | Flavan-3-ols | 255.03, 125.02, 227.04, 284.03, 285.04 |
| aa070 | Quercitrin | C_21_H_20_O_11_ | 448.1009 | 8.657 | Flavonol glycosides | 125.02, 143.05, 128.04, 137.02, 272.09 |
| aa071 | Luteolin | C_15_H_10_O_6_ | 286.0481 | 9.378 | Flavones | 167.03, 357.1, 387.11, 123.05, 125.02 |
| aa072 | Phloretin | C_15_H_14_O_5_ | 274.0845 | 9.534 | Others | 125.02, 169.01, 603.1, 177.02, 189.02 |
| aa073 | Oxalic acid | C_2_H_2_O_4_ | 89.99535 | 0.912 | Organic acids | 125.02, 169.01, 161.02, 271.03, 57.03 |
| aa074 | cis-Aconitic acid | C_6_H_6_O_6_ | 174.0165 | 1.019 | Organic acids | 125.02, 177.02, 255.03, 407.08, 161.02 |
| aa075 | L-Glutathione oxidized | C_20_H_32_N_6_O_12_S_2_ | 612.152 | 1.225 | Amino acid derivatives | 119.05, 151.04, 185.02, 93.03, 145.03 |
| aa076 | Guanine | C_5_H_5_N_5_O | 151.0495 | 1.234 | Nucleotide and its derivates | 96.96, 78.96, 96.05, 85.03, 96.97 |
| aa077 | Xanthosine | C_10_H_12_N_4_O_6_ | 284.0759 | 1.955 | Nucleotide and its derivates | 135.05, 133.05, 201.02, 134.04, 189.02 |
| aa078 | Esculin (6,7-Dihydroxycoumarin-6-glucoside) | C_15_H_16_O_9_ | 340.0797 | 4.729 | Coumarins | 361.09, 362.09, 147.04, 215.05, 197.04 |
| aa079 | Baimaside (Quercetin-3-O-sophoroside) | C_27_H_30_O_17_ | 626.149 | 7.266 | Flavonol glycosides | 135.05, 133.05, 201.02, 134.04, 189.02 |
| aa080 | Shikimic acid | C_7_H_10_O_5_ | 174.0527 | 6.26 | Organic acids | 153.02, 399, 154.02, 153.03, 224.95 |
| aa081 | Theacrine | C_9_H_12_N_4_O_3_ | 224.0908 | 5.625 | Alkaloids | 191.06, 75.01, 85.03, 179.06, 59.01 |
| aa082 | 1,2-di-O-galloyl-HHDP-glucose (Dalichasu) | C_34_H_26_O_22_ | 786.0927 | 6.964 | Glycosides | 153.02, 399, 154.02, 153.03, 224.95 |
| aa083 | GC-diGA | C_29_H_22_O_15_ | 610.0968 | 8.388 | Flavan-3-ols | 114.99, 130.98, 158.98, 116.99, 115.99 |
| aa084 | GC-GCG | C_37_H_30_O_18_ | 762.1441 | 5.721 | Flavan-3-ols | 88.99, 191.06, 78.96, 60.99, 85.03 |
| bb001 | trans-4-Hydroxy-L-proline | C_5_H_9_NO_3_ | 131.0583 | 1.267 | Amino acid derivatives | 213.03, 125.02, 167.03, 171.02, 195.02 |
| bb002 | Glutaric acid | C_5_H_8_O_4_ | 132.0423 | 1.851 | Organic acids | 125.02, 168.01, 124.02, 289.07, 109.03 |
| bb003 | 2,5-Dihydroxy benzoic acid O-hexside | C_13_H_16_O_9_ | 316.08 | 2.429 | Glycosides | 125.02, 463.22, 169.01, 71.01, 137.02 |
| bb004 | Ellagic acid glucoside | C_21_H_24_O_12_ | 468.1272 | 2.586 | Glycosides | 119.05, 163.04, 125.02, 135.05, 93.03 |
| bb005 | Protocatechuic acid O-glucoside | C_13_H_16_O_9_ | 316.08 | 2.934 | Glycosides | 135.05, 255.03, 191.06, 227.03, 173.09 |
| bb006 | 4-(beta-D-Glucosyloxy) benzoic acid | C_13_H_16_O_8_ | 300.0848 | 3.364 | Benzoic acid derivatives | 487.21, 487.08, 488.22, 488.09, 185.04 |
| bb007 | diGA-glucose | C_20_H_20_O_14_ | 484.0858 | 4.997 | Glycosides | 153.02, 163.04, 485.2, 317.08, 135.04 |
| bb008 | 3-O-p-Coumaroyl quinic acid | C_16_H_18_O_8_ | 338.1006 | 6.26 | Quinate and its derivatives | 255.03, 125.02, 227.04, 284.03, 285.04 |
| bb009 | Eriodictyol C-hexoside | C_21_H_22_O_11_ | 450.1168 | 6.265 | Flavanone glycosides | 147.04, 237.03, 131.05, 103.05, 90.98 |
| bb010 | Cyanidin 3-O-rutinoside (Keracyanin) | C_27_H_30_O_15_ | 594.159 | 8.587 | Anthocyanins | 125.02, 143.05, 128.04, 137.02, 272.09 |
| bb011 | diGC-GA | C_37_H_30_O_17_ | 746.1492 | 6.49 | Benzoic acid derivatives | 125.02, 177.02, 255.03, 407.08, 161.02 |
| bb012 | Nictoflorin | C_27_H_30_O_15_ | 594.159 | 7.903 | Flavonol glycosides | 167.03, 357.1, 387.11, 123.05, 125.02 |
| bb013 | 4',7-Dihydroxyflavanone | C_15_H_12_O_4_ | 256.0734 | 8.797 | Flavanones | 213.03, 125.02, 167.03, 171.02, 195.02 |
| cc001 | Agmatine | C_5_H_14_N_4_ | 130.1219 | 0.754 | Others | 147.04, 237.03, 131.05, 103.05, 90.98 |
| cc002 | Lactobionic acid | C_12_H_22_O_12_ | 358.1116 | 0.835 | Carbohydrates | 96.96, 78.96, 96.05, 85.03, 96.97 |
| cc003 | Nitrilacarb | C_9_H_15_N_3_O_2_ | 197.1165 | 0.839 | Others | 125.02, 169.01, 161.02, 271.03, 57.03 |
| cc004 | 1-Deoxy-D-altro-heptulose 7-phosphate | C_7_H_15_O_9_P | 274.0458 | 0.857 | Carbohydrates | 119.05, 163.04, 125.02, 135.05, 93.03 |
| cc005 | 4-Amino-4-deoxypentopyranose | C_5_H_11_NO_4_ | 149.0688 | 0.858 | Carbohydrates | 279.23, 78.96, 476.28, 280.24, 140.01 |
| cc006 | L-Pyroglutamic acid | C_5_H_7_NO_3_ | 129.0427 | 0.859 | Amino acid derivatives | 96.96, 125.02, 391.07, 79.96, 150 |
| cc007 | Maleamic acid | C_4_H_5_NO_3_ | 115.0269 | 0.868 | Organic acids | 135.05, 375.07, 191.06, 161.02, 88.99 |
| cc008 | (2S,4S)-4-Amino-2-hydroxy-2-methylpentanedioic acid | C_6_H_11_NO_5_ | 177.0637 | 0.874 | Organic acids | 184.07, 86.1, 520.34, 125, 104.11 |
| cc009 | Glycerol 3-phosphate | C_3_H_9_O_6_P | 172.0138 | 0.881 | Alcohols | 183.03, 124.02, 168.01, 125.02, 140.01 |
| cc010 | Glycerophosphoglycerol | C_6_H_15_O_8_P | 246.0506 | 0.882 | Alcohols | 78.96, 88.99, 153, 60.99, 125.02 |
| cc011 | 7-Hydroxy-6-methyl-8-(1-D-ribityl) lumazine | C_12_H_16_N_4_O_7_ | 328.1012 | 0.888 | Others | 125.02, 169.01, 150.03, 149.02, 80.97 |
| cc012 | 3-Phosphoglyceric acid | C_3_H_7_O_7_P | 185.9929 | 0.892 | Organic acids | 191.06, 85.03, 192.06, 93.03, 255.03 |
| cc013 | 3-Deoxy-D-manno-2-octulosonic acid | C_8_H_14_O_8_ | 238.0691 | 0.894 | Carbohydrates | 153.02, 425.14, 179.03, 255.06, 331.04 |
| cc014 | Uridine 5'-diphosphogalactose | C_15_H_24_N_2_O_17_P_2_ | 566.0558 | 0.899 | Nucleotide and its derivates | 125.02, 169.01, 150.03, 149.02, 80.97 |
| cc015 | 2-Acetamido-2-deoxyglucose | C_8_H_15_NO_6_ | 221.0899 | 0.903 | Carbohydrates | 411.27, 412.27, 409.16, 410.17, 413.28 |
| cc016 | Dihydrothymine | C_5_H_8_N_2_O_2_ | 128.0586 | 0.905 | Nucleotide and its derivates | 78.96, 88.99, 153, 60.99, 125.02 |
| cc017 | Trimethadione | C_6_H_9_NO_3_ | 143.0583 | 0.906 | Others | 119.05, 163.04, 125.02, 135.05, 93.03 |
| cc018 | Oxaceprol | C_7_H_11_NO_4_ | 173.0688 | 0.909 | Others | 507.07, 337.05, 508.08, 505.23, 506.23 |
| cc019 | L-Threonic acid | C_4_H_8_O_5_ | 136.0373 | 0.919 | Carbohydrates | 153.02, 467.19, 163.04, 131.05, 153.03 |
| cc020 | Hydroxycitric acid | C_6_H_8_O_8_ | 208.0221 | 0.928 | Organic acids | 361.09, 362.09, 147.04, 215.05, 197.04 |
| cc021 | Gly-L-pro | C_7_H_12_N_2_O_3_ | 172.0849 | 0.931 | Amino acid derivatives | 125.02, 169.01, 97.03, 273.08, 137.02 |
| cc022 | Ethanoic anhydride | C_4_H_6_O_3_ | 102.0317 | 0.932 | Organic acids | 78.96, 96.96, 96.97, 153, 409.24 |
| cc023 | Dihydrolipoic acid | C_8_H_16_O_2_S_2_ | 208.0585 | 0.934 | Vitamins | 153.02, 399, 154.02, 153.03, 224.95 |
| cc024 | alpha-Methyl D-mannoside | C_7_H_14_O_6_ | 194.0792 | 0.958 | Glycosides | 167.03, 357.1, 387.11, 123.05, 125.02 |
| cc025 | 4-Piperidinecarboxamide | C_6_H_12_N_2_O | 128.095 | 0.969 | Alkaloids | 78.96, 153, 191.06, 119.05, 96.97 |
| cc026 | Ethanal tetramer | C_8_H_16_O_4_ | 176.104 | 0.98 | Others | 213.03, 171.02, 125.02, 195.02, 108.05 |
| cc027 | Uridine monophosphate (UMP) | C_9_H_13_N_2_O_9_P | 324.0361 | 0.986 | Nucleotide and its derivates | 271.03, 300.03, 255.03, 463.09, 125.02 |
| cc028 | 2-Furoic acid | C_5_H_4_O_3_ | 112.0161 | 0.986 | Carbohydrates | 255.03, 227.04, 284.03, 417.08, 285.04 |
| cc029 | 5'-Xanthylic acid | C_10_H_13_N_4_O_9_P | 364.0413 | 1.005 | Nucleotide and its derivates | 135.05, 133.05, 201.02, 134.04, 189.02 |
| cc030 | N-Acetyl-L-glutamic acid | C_7_H_11_NO_5_ | 189.0638 | 1.069 | Amino acid derivatives | 78.96, 153, 171.01, 245.04, 96.97 |
| cc031 | Malonic acid | C_3_H_4_O_4_ | 104.011 | 1.075 | Organic acids | 411.27, 412.27, 409.16, 410.17, 413.28 |
| cc032 | Streptamine 4-phosphate | C_6_H_15_N_2_O_7_P | 258.0623 | 1.125 | Alcohols | 125.02, 168.01, 124.02, 289.07, 109.03 |
| cc033 | 2-Oxo-3-(5-oxofuran-2-ylidene)propanoic acid | C_7_H_4_O_5_ | 168.0062 | 1.152 | Organic acids | 487.21, 487.08, 488.22, 488.09, 185.04 |
| cc034 | L-Lupinic acid | C_13_H_18_N_6_O_3_ | 306.1426 | 1.222 | Organic acids | 183.03, 124.02, 168.01, 125.02, 140.01 |
| cc035 | Methyl 2-O-beta-L-arabinofuranosyl-beta-L-arabinofuranoside | C_11_H_20_O_9_ | 296.111 | 1.229 | Glycosides | 153.02, 163.04, 485.2, 317.08, 135.04 |
| cc036 | 2-(Carboxymethyl)-5-oxo-2,5-dihydro-2-furoic acid | C_7_H_6_O_6_ | 186.0165 | 1.229 | Amino acid derivatives | 131.05, 103.05, 132.05, 104.06, 407.33 |
| cc037 | Gly-Lys | C_8_H_17_N_3_O_3_ | 203.1272 | 1.234 | Amino acid derivatives | 96.96, 78.96, 96.05, 85.03, 96.97 |
| cc038 | (+)-a(S)-Butyr-amido-r-butyrolactone | C_8_H_13_NO_3_ | 171.0895 | 1.237 | Organic acids | 125.02, 169.01, 289.07, 109.03, 441.08 |
| cc039 | Prolylleucine | C_11_H_20_N_2_O_3_ | 228.1474 | 1.24 | Amino acid derivatives | 78.96, 96.97, 80.92, 96.96, 87.01 |
| cc040 | Guvacine | C_6_H_9_NO_2_ | 127.0634 | 1.291 | Vitamins | 125.02, 273.08, 97.03, 169.01, 255.07 |
| cc041 | 2-Methylcitric acid | C_7_H_10_O_7_ | 206.0427 | 1.304 | Organic acids | 135.05, 133.05, 201.02, 134.04, 189.02 |
| cc042 | 1,3,4-Trihydroxy-5-oxocyclohexanecarboxylic acid | C_7_H_10_O_6_ | 190.0479 | 1.321 | Organic acids | 255.03, 125.02, 227.04, 284.03, 285.04 |
| cc043 | DL-Mevalonic acid | C_6_H_12_O_4_ | 148.0736 | 1.353 | Organic acids | 167.04, 125.02, 123.01, 173.05, 153.02 |
| cc044 | Hymexazol O-glucoside | C_10_H_15_NO_7_ | 261.085 | 1.394 | Carbohydrates | 153.02, 467.19, 163.04, 131.05, 153.03 |
| cc045 | 5'-O-beta-D-Glucosylpyridoxine | C_14_H_21_NO_8_ | 331.1272 | 1.41 | Alcohols | 59.01, 71.01, 149.02, 89.02, 329.14 |
| cc046 | 3-Hydroxy-3-methylglutaric acid | C_6_H_10_O_5_ | 162.0529 | 1.417 | Organic acids | 125.02, 169.01, 211.02, 483.08, 93.03 |
| cc047 | N-Phenyl-beta-D-glucopyranosylamine | C_12_H_17_NO_5_ | 255.1106 | 1.489 | Carbohydrates | 147.04, 237.03, 131.05, 103.05, 90.98 |
| cc048 | N-Acetyl-L-2-aminoadipic acid | C_8_H_13_NO_5_ | 203.0796 | 1.507 | Amino acid derivatives | 119.05, 151.04, 185.02, 93.03, 145.03 |
| cc049 | alpha-Ketoadipic acid | C_6_H_8_O_5_ | 160.0372 | 1.596 | Organic acids | 125.02, 169.01, 211.02, 483.08, 93.03 |
| cc050 | tert-Butyl 3-amino-1-methyl-2,3-dioxopropylcarbamate | C_9_H_16_N_2_O_4_ | 216.1111 | 1.679 | Organic acids | 114.99, 130.98, 158.98, 116.99, 115.99 |
| cc051 | 2-(3,4,5-Trihydroxyphenyl)-3,4,5,7-chromanetetrol | C_15_H_14_O_8_ | 322.0693 | 1.749 | Flavonols | 331.07, 59.01, 125.02, 169.01, 123.01 |
| cc052 | (+/-)-2-Hydroxyglutaric acid | C_5_H_8_O_5_ | 148.0372 | 0.928 | Organic acids | 469.13, 147.04, 119.05, 470.14, 91.05 |
| cc053 | Lamiide | C_17_H_26_O_12_ | 422.1432 | 1.924 | Others | 135.05, 375.07, 191.06, 161.02, 88.99 |
| cc054 | Riboflavin reduced | C_15_H_16_N_4_O_6_ | 348.1061 | 2.063 | Vitamins | 125.02, 169.01, 603.1, 177.02, 189.02 |
| cc055 | Adenosine 5′-phosphoramidate | C_10_H_15_N_6_O_6_P | 346.0794 | 2.229 | Nucleotide and its derivates | 167.03, 357.1, 387.11, 123.05, 125.02 |
| cc056 | (Hydroxyethyl)methacrylate | C_6_H_10_O_3_ | 130.0631 | 2.439 | Organic acids | 437.19, 437.23, 438.2, 438.24, 439.2 |
| cc057 | trans-3-Indoleacrylic acid | C_11_H_9_NO_2_ | 187.0634 | 2.48 | Indole derivatives | 125.02, 169.01, 150.03, 149.02, 80.97 |
| cc058 | 3,5-Dihydroxy-4-[(6-O-sulfo-beta-D-glucopyranosyl)oxy]benzoic acid | C_13_H_16_O_13_S | 412.0317 | 2.528 | Benzoic acid derivatives | 411.27, 412.27, 409.16, 410.17, 413.28 |
| cc059 | Diacetin | C_7_H_12_O_5_ | 176.0685 | 2.825 | Organic acids | 135.05, 255.03, 191.06, 227.03, 173.09 |
| cc060 | 2,3-Dihydroxypropyl 3,4,5-trihydroxybenzoate | C_10_H_12_O_7_ | 244.0585 | 3.152 | Benzoic acid derivatives | 125.02, 273.08, 97.03, 169.01, 255.07 |
| cc061 | 3-[(1-Carboxyvinyl)oxy]benzoic acid | C_10_H_8_O_5_ | 208.0372 | 3.189 | Benzoic acid derivatives | 135.05, 133.05, 201.02, 134.04, 189.02 |
| cc062 | Malonylglycitin | C_25_H_24_O_13_ | 532.1223 | 3.198 | Amino acid derivatives | 271.03, 300.03, 255.03, 463.09, 125.02 |
| cc063 | Histidylglycine | C_8_H_12_N_4_O_3_ | 212.091 | 3.319 | Amino acid derivatives | 153.02, 399, 154.02, 153.03, 224.95 |
| cc064 | 5-[(E)-2-Carboxyvinyl]-2-hydroxyphenyl beta-D-glucopyranosiduronic acid | C_15_H_16_O_10_ | 356.0747 | 3.371 | Carbohydrates | 125.02, 169.01, 161.02, 271.03, 57.03 |
| cc065 | Diphenol glucuronide | C_12_H_14_O_8_ | 286.0691 | 3.588 | Carbohydrates | 167.04, 125.02, 123.01, 173.05, 153.02 |
| cc066 | Sulfanilic acid | C_6_H_7_NO_3_S | 173.0146 | 3.684 | Others | 211.02, 168.01, 169.01, 125.02, 505.06 |
| cc067 | Succinylacetone | C_7_H_10_O_4_ | 158.0579 | 3.693 | Organic acids | 139.04, 326.05, 307.08, 363.04, 140.04 |
| cc068 | Nifurquinazol | C_16_H_16_N_4_O_5_ | 344.1111 | 4.031 | Others | 125.02, 143.05, 128.04, 137.02, 272.09 |
| cc069 | beta-D-glucose pentaacetate | C_16_H_22_O_11_ | 390.1169 | 4.065 | Organic acids | 114.99, 130.98, 158.98, 116.99, 115.99 |
| cc070 | 5-(2-Carboxyethyl)-2-hydroxyphenyl beta-D-glucopyranosiduronic acid | C_15_H_18_O_10_ | 358.0904 | 4.085 | Carbohydrates | 119.05, 151.04, 185.02, 93.03, 145.03 |
| cc071 | 5'-S-Methyl-5'-thioadenosine | C_11_H_15_N_5_O_3_S | 297.0895 | 4.115 | Nucleotide and its derivates | 125.02, 169.01, 211.02, 483.08, 93.03 |
| cc072 | 1,5-Diphenylcarbazide | C_13_H_14_N_4_O | 242.1157 | 4.119 | Others | 290.08, 91.05, 310.6, 246.01, 74.1 |
| cc073 | 1,6-Bis-O-(3,4,5-trihydroxybenzoyl)hexopyranose | C_20_H_20_O_14_ | 484.0858 | 4.124 | Carbohydrates | 125.02, 169.01, 211.02, 483.08, 93.03 |
| cc074 | Zizybeoside I | C_19_H_28_O_11_ | 432.1638 | 4.147 | Coumarins | 78.96, 88.99, 153, 60.99, 125.02 |
| cc075 | (1S)-1,5-Anhydro-1-[2,4,6-trihydroxy-3-(4-hydroxybenzoyl)phenyl]-D-glucitol | C_19_H_20_O_10_ | 408.1063 | 4.222 | Alcohols | 78.96, 96.96, 96.97, 153, 409.24 |
| cc076 | (-)-L-Chicoric acid | C_22_H_18_O_12_ | 474.0802 | 4.254 | Organic acids | 167.04, 125.02, 123.01, 173.05, 153.02 |
| cc077 | N-Acetyl-D-alloisoleucine | C_8_H_15_NO_3_ | 173.1054 | 4.291 | Amino acid derivatives | 125.02, 463.22, 169.01, 71.01, 137.02 |
| cc078 | Melilotoside | C_15_H_18_O_8_ | 326.1006 | 4.301 | Organic acids | 191.06, 85.03, 192.06, 93.03, 255.03 |
| cc079 | Phenylacetaldehyde | C_8_H_8_O | 120.0574 | 4.308 | Benzoic acid derivatives | 78.96, 96.97, 80.92, 96.96, 87.01 |
| cc080 | 4-(2-Carboxyethyl)-2-methoxyphenyl beta-D-glucopyranosiduronic acid | C_16_H_20_O_10_ | 372.106 | 4.336 | Carbohydrates | 191.06, 75.01, 85.03, 179.06, 59.01 |
| cc081 | 5-Hydroxy-6-methoxy-3-(4-methoxyphenyl)-4-oxo-4H-chromen-7-yl beta-D-glucopyranosiduronic acid | C_23_H_22_O_12_ | 490.1118 | 4.405 | Carbohydrates | 183.03, 124.02, 168.01, 125.02, 140.01 |
| cc082 | Aspirin | C_9_H_8_O_4_ | 180.0423 | 4.422 | Organic acids | 271.03, 300.03, 255.03, 463.09, 125.02 |
| cc083 | Propentofylline | C_15_H_22_N_4_O_3_ | 306.169 | 4.61 | Alkaloids | 211.02, 168.01, 169.01, 125.02, 505.06 |
| cc084 | 1-O-vanilloyl-beta-D-glucose | C_14_H_18_O_9_ | 330.0955 | 4.724 | Carbohydrates | 125.02, 167.04, 209.05, 123.05, 239.06 |
| cc085 | Benzyl 6-O-beta-D-xylopyranosyl-beta-D-glucopyranoside | C_18_H_26_O_10_ | 402.1531 | 4.726 | Carbohydrates | 88.99, 191.06, 78.96, 60.99, 85.03 |
| cc086 | 1,3-Propane sultone | C_3_H_6_O_3_S | 122.0038 | 4.746 | Others | 153.02, 425.14, 179.03, 255.06, 331.04 |
| cc087 | 7-Ethoxycoumarin | C_11_H_10_O_3_ | 190.063 | 4.766 | Coumarins | 125.02, 143.05, 128.04, 137.02, 272.09 |
| cc088 | Sinapaldehyde glucoside | C_17_H_22_O_9_ | 370.1269 | 4.768 | Carbohydrates | 153.02, 399, 154.02, 153.03, 224.95 |
| cc089 | Kynurenic acid | C_10_H_7_NO_3_ | 189.0426 | 4.787 | Coumarins | 59.01, 71.01, 149.02, 89.02, 329.14 |
| cc090 | Caffeoylmalic acid | C_13_H_12_O_8_ | 296.053 | 4.942 | Organic acids | 139.04, 326.05, 307.08, 363.04, 140.04 |
| cc091 | 2,4-Diacetylphloroglucinol | C_10_H_10_O_5_ | 210.0529 | 5.238 | Others | 191.06, 75.01, 85.03, 179.06, 59.01 |
| cc092 | 1-Naphthyl glucuronide | C_16_H_16_O_7_ | 320.0897 | 5.29 | Carbohydrates | 184.07, 86.1, 520.34, 125, 104.11 |
| cc093 | 5-Nitro-2-propoxyaniline | C_9_H_12_N_2_O_3_ | 196.0845 | 6.262 | Others | 125.02, 169.01, 603.1, 177.02, 189.02 |
| cc094 | 5-(3,4-diacetoxybut-1-ynyl)-2,2'-bithiophene | C_16_H_14_O_4_S_2_ | 334.0331 | 6.322 | Others | 78.96, 153, 171.01, 245.04, 96.97 |
| cc095 | 3-(5'-methylthio)pentylmalic acid | C_10_H_18_O_5_S | 250.0877 | 6.323 | Organic acids | 96.96, 125.02, 391.07, 79.96, 150 |
| cc096 | Gentiopicrin | C_16_H_20_O_9_ | 356.1108 | 6.402 | Glycosides | 125.02, 177.02, 255.03, 407.08, 161.02 |
| cc097 | 1,5-Anhydro-1-[3-(3,4-dihydroxybenzoyl)-2,4,6-trihydroxyphenyl]hexitol | C_19_H_20_O_11_ | 424.0988 | 6.45 | Alcohols | 153.02, 425.14, 179.03, 255.06, 331.04 |
| cc098 | Sweroside | C_16_H_22_O_9_ | 358.127 | 6.451 | Terpenes | 135.05, 133.05, 201.02, 134.04, 189.02 |
| cc099 | 4,7,8-Trihydroxy-3-(4-hydroxyphenyl)dibenzo[b,d]furan-1,2-diyl diacetate | C_22_H_16_O_9_ | 424.0793 | 6.952 | Organic acids | 78.96, 96.97, 80.92, 96.96, 87.01 |
| cc100 | N-Acetyl-DL-tryptophan | C_13_H_14_N_2_O_3_ | 246.1006 | 6.973 | Amino acid derivatives | 78.96, 153, 191.06, 119.05, 96.97 |
| cc101 | 1-(β-D-Glucopyranosyloxy)-7-methyl-1,4a,5,6,7,7a-hexahydrocyclopenta[c]pyran-4-carboxylic acid | C_16_H_24_O_9_ | 360.1426 | 7.035 | Organic acids | 361.09, 362.09, 147.04, 215.05, 197.04 |
| cc102 | 1,6-bis-O-galloyl-beta-D-glucose | C_20_H_20_O_14_ | 484.0854 | 7.267 | Carbohydrates | 125.02, 169.01, 211.02, 483.08, 93.03 |
| cc103 | 8-Demethyl-8-(alpha-L-rhamnosyl)tetracenomycin C | C_28_H_28_O_15_ | 604.1438 | 7.41 | Glycosides | 125.02, 169.01, 603.1, 177.02, 189.02 |
| cc104 | iso-Quercitrin 6"-acetate | C_23_H_22_O_13_ | 506.1068 | 7.624 | Flavonol glycosides | 211.02, 168.01, 169.01, 125.02, 505.06 |
| cc105 | 4-Carboxy nevirapine | C_15_H_12_N_4_O_3_ | 296.0898 | 7.679 | Others | 125.02, 169.01, 211.02, 483.08, 93.03 |
| cc106 | Mascaroside | C_26_H_36_O_11_ | 524.2264 | 7.785 | Terpenes | 331.07, 59.01, 125.02, 169.01, 123.01 |
| cc107 | Glucosylgalactosyl hydroxylysine | C_18_H_34_N_2_O_13_ | 486.2077 | 7.791 | Amino acid derivatives | 487.21, 487.08, 488.22, 488.09, 185.04 |
| cc108 | beta-D-Fructofuranosyl 2,4-bis-O-(3-methylbutanoyl)-alpha-D-glucopyranoside | C_22_H_38_O_13_ | 510.2317 | 7.792 | Carbohydrates | 125.02, 463.22, 169.01, 71.01, 137.02 |
| cc109 | Eugenyl Glucoside | C_16_H_22_O_7_ | 326.1365 | 7.814 | Terpenes | 131.05, 103.05, 132.05, 104.06, 407.33 |
| cc110 | beta-Syringin | C_17_H_24_O_9_ | 372.1424 | 7.823 | Hydroxycinnamoyl derivatives | 153.02, 399, 154.02, 153.03, 224.95 |
| cc111 | Astilbin | C_21_H_22_O_11_ | 450.1169 | 7.902 | Flavonol glycosides | 213.03, 125.02, 167.03, 171.02, 195.02 |
| cc112 | Malonylgenistin | C_24_H_22_O_13_ | 518.1071 | 8.088 | Isoflavone glycosides | 213.03, 171.02, 125.02, 195.02, 108.05 |
| cc113 | 11-O-Demethyl-7-methoxypradinone II | C_25_H_18_O_11_ | 494.0832 | 8.096 | Others | 125.02, 169.01, 97.03, 273.08, 137.02 |
| cc114 | Gallocatechin-(4alpha->8)-epigallocatechin | C_30_H_26_O_14_ | 610.132 | 8.303 | Anthocyanins | 125.02, 169.01, 161.02, 271.03, 57.03 |
| cc115 | N-Adenylylanthranilic acid | C_17_H_19_N_6_O_8_P | 466.1018 | 8.383 | Benzoic acid derivatives | 153.02, 467.19, 163.04, 131.05, 153.03 |
| cc116 | Eupatoriochromene | C_13_H_14_O_3_ | 218.0943 | 8.49 | Others | 88.99, 191.06, 78.96, 60.99, 85.03 |
| cc117 | Tiliroside | C_30_H_26_O_13_ | 594.1375 | 8.527 | Flavonol glycosides | 255.03, 125.02, 227.04, 284.03, 285.04 |
| cc118 | Olsalazine | C_14_H_10_N_2_O_6_ | 302.0538 | 8.64 | Organic acids | 125.02, 169.01, 289.07, 109.03, 441.08 |
| cc119 | (2Z)-3-[4,5-Dihydroxy-2-(2-hydroxy-2-propanyl)-2,3-dihydro-1-benzofuran-7-yl]acrylic acid | C_14_H_16_O_6_ | 280.0949 | 8.717 | Organic acids | 469.13, 147.04, 119.05, 470.14, 91.05 |
| cc120 | Cynarine | C_25_H_24_O_12_ | 516.1273 | 8.772 | Quinate and its derivatives | 135.05, 255.03, 191.06, 227.03, 173.09 |
| cc121 | (2R,3R)-5,7-Dihydroxy-2-(4-hydroxyphenyl)-3,4-dihydro-2H-chromen-3-yl 3,4,5-trihydroxybenzoate | C_22_H_18_O_9_ | 426.0956 | 8.8 | Benzoic acid derivatives | 125.02, 273.08, 97.03, 169.01, 255.07 |
| cc122 | Azelaic acid | C_9_H_16_O_4_ | 188.1049 | 8.832 | Organic acids | 125.02, 167.04, 209.05, 123.05, 239.06 |
| cc123 | Swertianolin | C_20_H_20_O_11_ | 436.101 | 8.835 | Others | 96.96, 125.02, 391.07, 79.96, 150 |
| cc124 | Salvianolic acid D | C_20_H_18_O_10_ | 418.0904 | 8.849 | Organic acids | 255.03, 227.04, 284.03, 417.08, 285.04 |
| cc125 | Luteolin 7-O-(6-O-malonyl-beta-D-glucoside) | C_24_H_22_O_14_ | 534.1014 | 8.865 | Flavone glycosides | 191.06, 85.03, 192.06, 93.03, 255.03 |
| cc126 | Picrotin | C_15_H_18_O_7_ | 310.1056 | 8.876 | Others | 507.07, 337.05, 508.08, 505.23, 506.23 |
| cc127 | (-)-Caryophyllene oxide | C_15_H_24_O | 220.1828 | 8.894 | Others | 78.96, 96.96, 96.97, 153, 409.24 |
| cc128 | Naringin dihydrochalcone | C_27_H_34_O_14_ | 582.1959 | 9.006 | Flavanone glycosides | 125.02, 167.04, 209.05, 123.05, 239.06 |
| cc129 | Amarogentin | C_29_H_30_O_13_ | 586.1696 | 9.201 | Terpenes | 59.01, 71.01, 149.02, 89.02, 329.14 |
| cc130 | Silandrin | C_25_H_22_O_9_ | 466.126 | 9.243 | Flavanones | 151.04, 151, 115, 133.01, 71.01 |
| cc131 | Lappaol | C_30_H_34_O_10_ | 554.2162 | 9.317 | Others | 131.05, 103.05, 132.05, 104.06, 407.33 |
| cc132 | Kolaflavanone | C_31_H_24_O_12_ | 588.1278 | 9.335 | Flavanones | 78.96, 153, 171.01, 245.04, 96.97 |
| cc133 | Atrovirinone | C_25_H_28_O_8_ | 456.1792 | 9.363 | Others | 125.02, 168.01, 124.02, 289.07, 109.03 |
| cc134 | Lamifiban | C_24_H_28_N_4_O_6_ | 468.1999 | 9.385 | Others | 469.13, 147.04, 119.05, 470.14, 91.05 |
| cc135 | Chitobiose | C_16_H_28_N_2_O_11_ | 424.1707 | 9.407 | Carbohydrates | 290.08, 91.05, 310.6, 246.01, 74.1 |
| cc136 | 2-Amino-1,3,4-octadecanetriol | C_18_H_39_NO_3_ | 317.2929 | 9.547 | Alcohols | 213.03, 171.02, 125.02, 195.02, 108.05 |
| cc137 | Methyl 3,4,5-trimethoxycinnamate | C_13_H_16_O_5_ | 252.1 | 9.603 | Hydroxycinnamoyl derivatives | 437.19, 437.23, 438.2, 438.24, 439.2 |
| cc138 | Traumatic acid | C_12_H_20_O_4_ | 228.1364 | 9.612 | Organic acids | 135.05, 375.07, 191.06, 161.02, 88.99 |
| cc139 | Propofol | C_12_H_18_O | 178.1358 | 9.693 | Others | 331.07, 59.01, 125.02, 169.01, 123.01 |
| cc140 | 7-Dehydrocholesterol benzoate | C_34_H_48_O_2_ | 488.366 | 9.784 | Hydroxycinnamoyl derivatives | 125.02, 169.01, 289.07, 109.03, 441.08 |
| cc141 | Demethylphylloquinone | C_30_H_44_O_2_ | 436.3342 | 9.815 | Others | 437.19, 437.23, 438.2, 438.24, 439.2 |
| cc142 | 1,4-Anhydro-6-O-dodecanoyl-2,3-bis-O-(2-hydroxyethyl)-D-glucitol | C_22_H_42_O_8_ | 434.2883 | 10.011 | Alcohols | 78.96, 153, 191.06, 119.05, 96.97 |
| cc143 | Embelin | C_17_H_26_O_4_ | 294.1835 | 10.082 | Alcohols | 279.23, 78.96, 476.28, 280.24, 140.01 |
| cc144 | 2-Linoleoyl-sn-glycero-3-phosphoethanolamine | C_23_H_44_NO_7_P | 477.2859 | 10.199 | Others | 279.23, 78.96, 476.28, 280.24, 140.01 |
| cc145 | Matricin | C_17_H_22_O_5_ | 306.147 | 10.23 | Terpenes | 125.02, 169.01, 97.03, 273.08, 137.02 |
| cc146 | Mucronine A | C_29_H_38_N_4_O_4_ | 506.2888 | 10.375 | Alkaloids | 507.07, 337.05, 508.08, 505.23, 506.23 |
| cc147 | 1-Oleoyl-sn-glycero-3-phosphoethanolamine | C_23_H_46_NO_7_P | 479.3016 | 10.377 | Others | 139.04, 326.05, 307.08, 363.04, 140.04 |
| cc148 | 1-Linoleoyl-sn-glycero-3-phosphocholine | C_26_H_50_NO_7_P | 519.3326 | 10.47 | Others | 184.07, 86.1, 520.34, 125, 104.11 |
| cc149 | Lauramide | C_12_H_25_NO | 199.1936 | 10.698 | Others | 125.02, 177.02, 255.03, 407.08, 161.02 |
| cc150 | Allylcyclohexane | C_9_H_16_ | 124.1253 | 11.966 | Others | 290.08, 91.05, 310.6, 246.01, 74.1 |
| cc151 | p-Menth-3-ene | C_10_H_18_ | 138.1409 | 11.966 | Others | 151.04, 151, 115, 133.01, 71.01 |
| cc152 | 4,5-Dihydroxy-4-(2,3,4-trihydroxytetrahydro-2-furanyl)-3,4-dihydro-2H-pyrrole-2-carboxylic acid | C_9_H_13_NO_8_ | 263.0643 | 0.92 | Indole derivatives | 151.04, 151, 115, 133.01, 71.01 |
| cc153 | beta-D-Ethyl glucuronide | C_8_H_14_O_7_ | 222.0743 | 0.922 | Carbohydrates | 255.03, 227.04, 284.03, 417.08, 285.04 |
| cc154 | 7-Hydroxycoumarine | C_9_H_6_O_3_ | 162.0317 | 5.189 | Coumarins | 153.02, 163.04, 485.2, 317.08, 135.04 |
